# Multifunctional Nano Immunostimulant: Overcoming Immunosuppressive Microenvironment for Antitumor Immunotherapy

**DOI:** 10.1002/advs.202517480

**Published:** 2025-12-12

**Authors:** Guanhong Guo, Wenda Zhong, Huishuang Zhao, Yueying An, Xinyu Dong, Zhengbo Li, Shuangfeng Qin, Guangzhao Xu, Xiangguo Yue, Xudong Wang, Wen Sun, Zhe‐Sheng Chen, Weiguo Song, Liuya Wei, Fahui Li

**Affiliations:** ^1^ School of Pharmacy Shandong Second Medical University Weifang 261053 P. R. China; ^2^ Weifang University of Science and Technology Weifang 262700 P. R. China; ^3^ Harway Pharma (Weifang) Co., Ltd Weifang 262700 P. R. China; ^4^ State Key Laboratory of Fine Chemicals Dalian University of Technology Dalian 116024 P. R. China; ^5^ Department of Pharmaceutical Sciences College of Pharmacy and Health Sciences St. John's University New York NY 11439 USA

**Keywords:** antitumor immunotherapy, ferroptosis, immunogenic cell death, immunosuppressive microenvironment

## Abstract

Antitumor immunotherapy has become a pillar therapy by activating the immune system to recognize and attack tumor cells. Yet its efficacy is limited by the immunosuppressive tumor microenvironment (TME) and related mechanisms like hypoxia, high glutathione (GSH) expression, and immune evasion. Due to TME complexity and tumor heterogeneity, monotherapy struggles to modulate immunosuppressive factors for potent results. To solve this, this work develops a multifunctional immune stimulator (3IZH), which can simultaneously boost immunity, downregulate GSH, and alleviate hypoxia. In weakly acidic TME, it releases photosensitizer (3ICy5) and Fe ions. Fe ions consume GSH and relieve hypoxia via redox reactions and hydrogen peroxide decomposition. 3ICy5 accumulates in the endoplasmic reticulum (ER), produces ROS, induces severe ER stress and DAMPs release, triggering immunogenic cell death (ICD). Fe ions and ROS also reduce glutathione peroxidase 4 (GPX4), causing ferroptosis. ICD and ferroptosis activate T cell infiltration to restructure TME. Combined with HIF‐1α inhibitor digoxin, 3IZH further reduces HIF‐1α resistance, enhances immune cell infiltration, and shows satisfying efficacy in bilateral tumor‐bearing mice. The regulatory effect of the immune‐suppressive TME, the remarkable therapeutic effect, as well as the safety profile, together indicate the potential of the multifunctional immune stimulator design strategy.

## Graphical abstract

A multifunctional nano immunostimulant is first proposed for efficient tumor immunotherapy. The ER targeted ability, superior ROS production, efficient GSH consumption, coupled with the HIF suppression, jointly arouses ICD/ferroptosis pathways to realize efficient tumor immunotherapy. This work provides a new design strategy for simultaneously promoting antigen presentation and reconstructing the immunosuppressive TME.

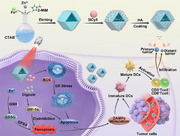

## 1 Introduction

Anti‐tumor immunotherapy, which activates or enhances the immune system to recognize and attack tumor cells, has become one of the pillar tumor therapies, like surgery, radiotherapy, and chemotherapy.^[^ ^1^, ^2^ ^]^ Despite significant progress, immunotherapy is still limited by the complexity of the TME and challenges related to immune suppression mechanisms, such as hypoxia, high GSH expression, and immune suppression.^[^ ^3^, ^4^, ^5^, ^6^ ^]^ In the hypoxic TME, the HIF‐1α pathway is activated, promoting the secretion of immune suppressive factors (such as VEGF and IL‐10), weakening Tregs (T) cell infiltration and function, and leading to immune evasion.^[^ ^7^, ^8^ ^]^ Moreover, the increased HIF‐1α will resist the ferroptosis and apoptosis related immunotherapy.^[^ ^9^, ^10^, ^11^ ^]^ High GSH expression can enhance the antioxidant capacity of tumor cells, neutralize lipid peroxides, and weaken immune killing mechanisms dependent on oxidative stress, such as ferroptosis. Additionally, the tumor microenvironment induces T cell exhaustion or the secretion of factors like TGF‐β by regulatory T cells and myeloid‐derived suppressor cells (MDSCs) through PD‐L1 overexpression, suppressing the activity of immune cells.^[^ ^12^, ^13^ ^]^ Innovative therapies that address the challenges of hypoxia, high GSH expression, and immune suppression through tumor microenvironment remodeling and achieve effective anti‐tumor immunity are still urgently needed.

To address the issue of hypoxia, clinical treatments employ hyperbaric oxygen therapy, inhalation of carbon‐oxygen mixed gas, and the use of drugs to improve oxygen delivery. To reduce the expression of GSH in the TME, researchers have designed a series of responsive nanocarriers and chemical bonds to downregulation GSH. For example, by utilizing the reducing property of GSH, researchers have developed a series of nanocarrier drug delivery systems that can be used to reduce GSH levels. They can also use BSO, a drug that inhibits γ‐glutamyl cysteine synthase (the rate‐limiting enzyme for GSH synthesis), to block the biosynthetic pathway of GSH.^[^ ^14^, ^15^ ^]^ To activate immune cell populations, researchers have developed various therapeutic strategies, the most common of which are immune checkpoint blockade (e.g., PD‐1/PD‐L1 inhibitors), TME modulation to alleviate immunosuppression (e.g., anti‐CCR4 antibodies for regulatory T cell depletion) and epigenetic reprogramming of immune responses (e.g., Chidamide‐induced MHC‐I upregulation to enhance antigen presentation).^[^ ^16^, ^17^, ^18^, ^19^ ^]^ However, the complexity and heterogeneity of TME often lead to acquired resistance to mono‐therapy, making it challenging to effectively overcome immunosuppression and achieve efficient antitumor immunity.

It has been reported that photodynamic‐based immune stimulator can effectively activates anti‐tumor immunity and counteract immunosuppression within the TME by promoting various forms of inflammatory cell death pathways.^[^ ^20^ ^]^ However, the conventional photodynamic monotherapy consumes oxygen, exacerbating hypoxia and subsequently upregulating HIF expression, which will promote tumor invasion and metastasis.^[^ ^21^ ^]^ Therefore, developing a photodynamic‐based combinatorial strategy that simultaneously addresses hypoxia, GSH overexpression, and immunosuppression represents a feasible approach for achieving potent antitumor immunotherapy. This integrated therapeutic paradigm aims to overcome multiple biological barriers. Hypoxia mitigation through applying oxygen‐economizing photodynamic immune stimulator, enhancing oxygen supply and inhibiting the HIF expression. GSH depletion via redox‐modulating agents and amplifying oxidative stress. Immune reactivation by reversing T cell anergy and Treg infiltration. Thus, the key challenges include optimizing spatiotemporal control and release of photodynamic immune stimulator, GSH inhibitor, and HIF alleviator. Moreover, monitoring the immune‐mediated effects and elucidating corresponding mechanisms remains challenging.

Herein, a multifunctional immune stimulator 3IZH is designed. The pH‐responsive Fe‐doped ZIF‐8 acted as the carrier, which can release the Fe ions in the acidic TME for consuming GSH and relieving hypoxia. The efficient photodynamic immune arouser 3ICy5 can be released simultaneously with Fe ions. Due to the ER targeting and ROS generation ability, 3ICy5 can activate the ICD pathway. The release of Fe ions and ROS generation will simultaneously consume the GSH, eventually down‐regulate GPX4 and further activate the ferroptosis pathway. The co‐activated inflammatory pathways of ICD and ferroptosis promote the release of DAMPs, which activate and proliferate the infiltration of antigen‐specific T cells, eventually realizing the efficient anti‐tumor immunotherapy. The further application of HIF‐1α inhibitor digoxin with 3IZH effectively reduced the resistance of HIF to ferroptosis and apoptosis. Thus, in the bilateral tumor‐bearing mice models, the combination application of digoxin with 3IZH showed satisfying performance in inhibiting the growth of the distant and proximal tumors. The regulatory effect of the immune‐suppressive TME, the remarkable therapeutic effect, as well as the safety profile, together indicate the potential of the multifunctional immune stimulator design strategy.

## 2 Results and Discussion

### 2.1 Preparation, Characterization, and In Vitro ROS Generation of 3IZH

**Figure 1:**
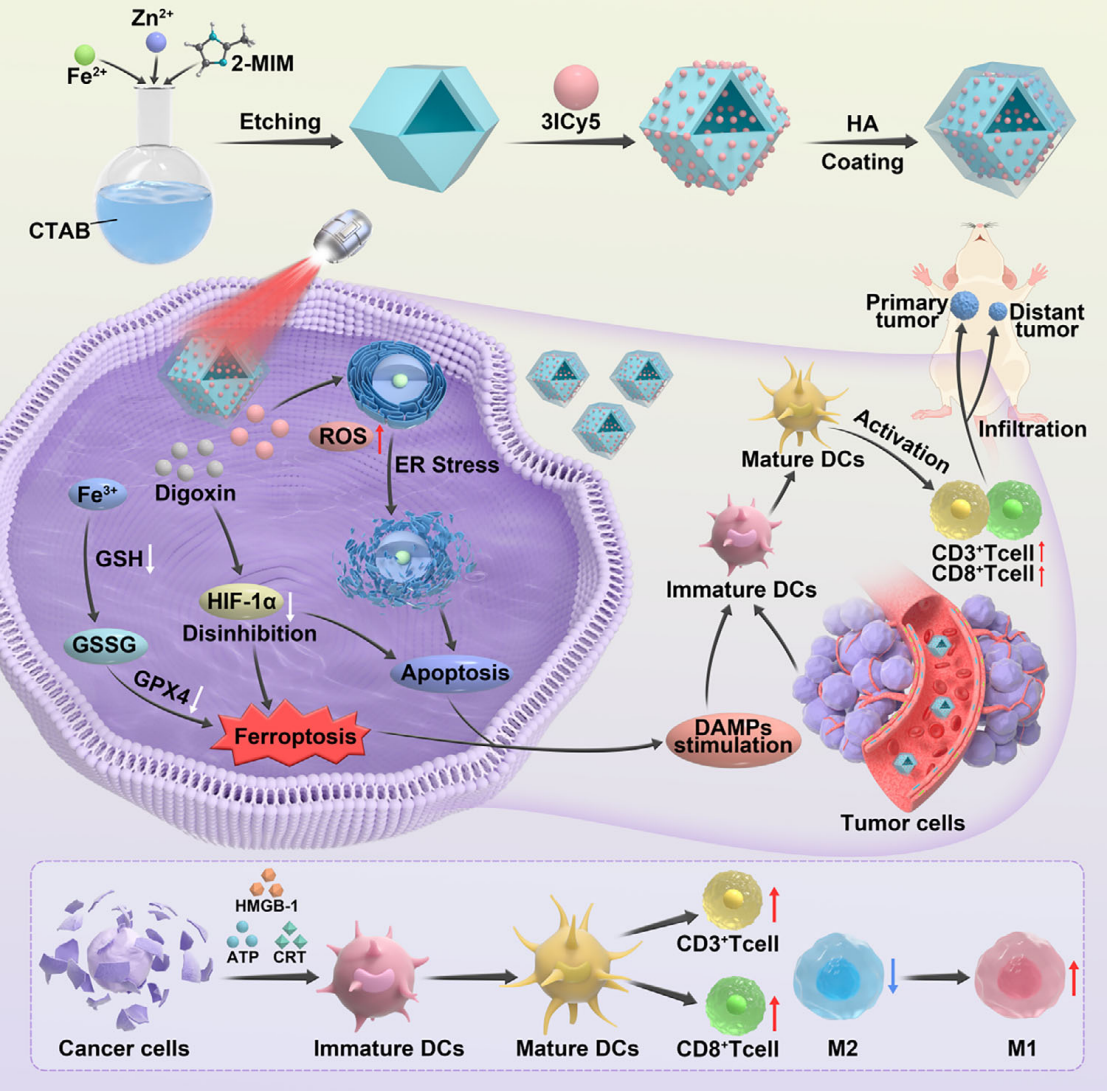
Schematic diagram of 3IZH preparation and antitumor immunotherapy mechanism by combining 3IZH with digoxin.

**Figure** **1** provides a detailed illustration of the design strategy for 3IZH nanoparticles. ZnFe‐ZIF was synthesized in an aqueous solution of cetyltrimethylammonium bromide (CTAB) using a mixture of FeCl_3_ and 2‐methylimidazole (2‐MIM). Tannic acid (TA) was then used to chemically etch ZnFe‐ZIF, resulting in the formation of hollow mesoporous ZnFe‐ZIF. 3ICy5 was efficiently loaded onto ZnFe‐ZIF via capillary adsorption, yielding the 3IZ complex. Subsequently, hyaluronic acid (HA) was uniformly encapsulated on the exterior of 3IZ, resulting in the formation of the final 3IZH nanoparticles. In this design, the coating with HA can enhance the accumulation of the 3IZH nanoparticles in the tumor tissue. Meanwhile, the negative charge of TA can enhance the stability of the 3IZH nanoparticles in the circulatory system, ensuring that there is sufficient nanodrug that can effectively remain in the tumor tissue. When the 3IZH nanoparticles are taken into the cell by endocytosis, the Fe^3+^ released from FeZn‐ZIF can respond to the GSH levels in the TME, reducing intracellular GSH and inhibiting the antioxidant function of GPX4, thereby promoting ferroptosis in tumor cells. Moreover, under NIR irradiation, the released 3ICy5 can induce ER stress and reduce the GSH by generating abundant ROS, eventually boosting the DAMPs release for the ICD pathway. This process eventually promotes the infiltration of dendritic cells (DCs) and T lymphocytes. After the combination of digoxin, the inhibitory effect of HIF‐1α on ferroptosis will be mitigated, which further ensures the ferroptosis process for efficient anti‐tumor immunotherapy. The detailed information regarding the synthesis and characterization of 3ICy5 can be referred to Scheme S1 and Figures S1–S5 (Supporting Information).

**Figure 2:**
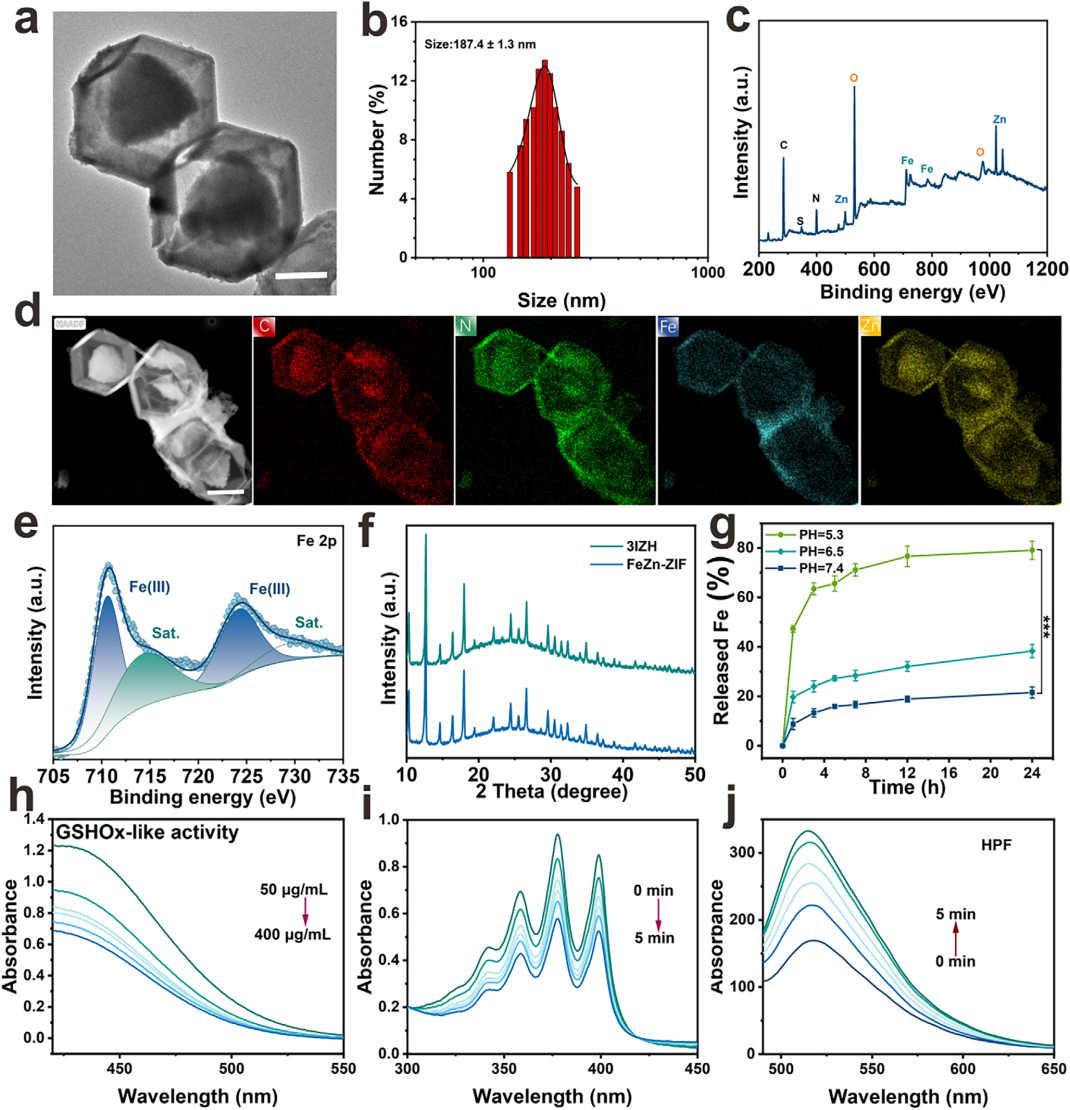
a) TEM image of 3IZH; scar bar = 50 nm. b) The nanoparticle size of 3IZH. c) XPS survey spectrum of 3IZH. d) Corresponding elemental mapping of 3IZH; scar bar = 100 nm. e) Fe 2p spectrum of 3IZH. f) XRD survey spectra of FeZn‐ZIF and 3IZH. g) Fe‐released profile of 3IZH at different pH values. h) DTNB probe caused by 3IZH under acidic conditions for GSH depletion detection. i) Detection of ^1^O_2_ generation using ABDA assay under 660 nm laser irradiation (1 W cm^−2^). j) ROS generation under NIR light irradiation (660 nm, 10 mW cm^−2^) after co‐incubation of HPF with 3IZH. (mean ± SD, n = 3, *p < 0.05, **p < 0.01, and ***p < 0.001). One‐way analysis of variance (ANOVA) was used to assess significance.

Transmission electron microscopy (TEM) analysis demonstrated that the synthesized 3IZH nanoparticles exhibit a polyhedral morphology similar to that of ZIF‐8, with a hollow structure (**Figure** **2**a). The positively charged surface of 3IZ nanoparticles spontaneously and firmly binds to the negatively charged HA during incubation, while the Zeta potential remains consistently negative after serum incubation (Figure S6, Supporting Information). This stability is crucial for the nanoparticles to evade the membrane penetration system and achieve effective tumor accumulation via the EPR effect. Dynamic light scattering (DLS) measurements indicated an average size of 187.4 ± 1.3 nm for 3IZH (Figure 2b). The X‐ray photoelectron spectroscopy (XPS) analysis (Figure 2c) and elemental mapping analysis (Figure 2d) co‐indicated that the 3IZH nanoparticles contained N, C, Fe, and Zn. According to the previous report, the binding energy values of Fe 2p3/2 and Sat.1 peaks at about 710 and 715 eV can be used to confirm that the oxidation state of Fe ions in 3IZH is Fe^3+^ (Figure 2e). UV–vis absorption emission spectra further verified the successful loading of 3ICy5 into the FeZn‐ZIF matrix (Figure S7, Supporting Information). X‐ray powder diffraction (XRD) measurement showed that the crystal structure of FeZn‐ZIF remained unchanged after the incorporation of 3ICy5 (Figure 2f).

To investigate the pH‐dependent release profile of 3IZH, the nanoparticles were incubated in phosphate‐buffered saline (PBS) at various pH values. Under physiological pH of 7.4, as shown in Figure 2g and Figure S8 (Supporting Information), 3IZH exhibits excellent stability, with only 21.5% of Fe^3+^ and 20.1% of 3ICy5 being released over a 24‐hour period. In contrast, when subjected to an acidic environment at pH 5.3, over 70% of Fe^3+^ and 3ICy5 in 3IZH nanoparticles were liberated after 24 hours, highlighting the strong pH‐responsive properties of 3IZH. Given that the overexpression of GSH concentration (≈10 mm) in tumors can effectively scavenge ROS, 5,5′‐dithiobis(2‐nitrobenzoic acid) (DTNB) was employed as a GSH indicator to assess the consumption capability of 3IZH on GSH. DTNB reacted with GSH to generate a detectable colored product. In the mixed system of GSH and DTNB, as the concentration of 3IZH increased, the UV–visible absorbance at 412 nm significantly decreased, further confirming that 3IZH exhibited a notable capacity to consume GSH (Figure 2h). Subsequently, the ability of 3IZH to generate ^1^O_2_ under NIR light irradiation (for 5 min) was assessed using ABDA, and the results demonstrated that 3IZH exhibited excellent ^1^O_2_ generation capability (Figure 2i). Additionally, experiments utilizing HPF and DHR123 probes further confirmed the effective type I ROS generation capability of 3IZH under NIR light irradiation (Figure 2j; Figure S9, Supporting Information). These results underscore the stability and clinical translation potential of 3IZH nanoparticles.

### 2.2 In Vitro Induction of ER Stress and Immunogenic Cell Death

**Figure 3:**
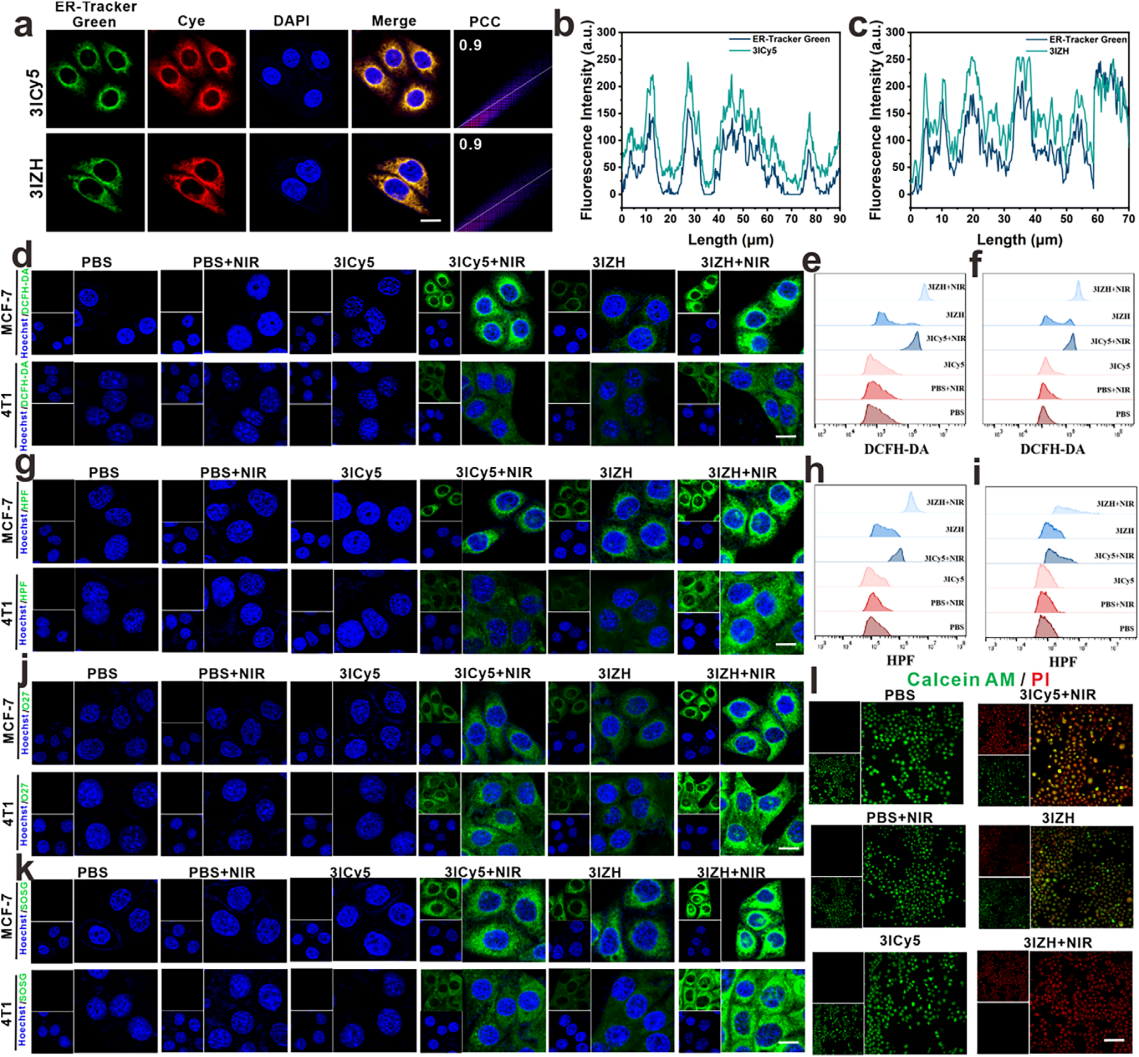
Spontaneous ER‐localizability of 3ICy5 and 3IZH. a) Representative merged CLSM images of MCF7 cells treated with 0.5 µM of ER‐Tracker Green and 1.8 µg mL^−1^ of 3ICy5 and 1.2 µg mL^−1^ 3IZH (red) for 2 h. Scale bar = 10 µm. b,c) Pearson correlation coefficients of the 3ICy5 and 3IZH/ER‐Tracker Green signal overlap in the CLSM images. Insets (d), (g), (j), and (k) represent the fluorescence intensities of relevant indicators of cells treated with 3ICy5 or 3IZH after NIR irradiation (660 nm, 1 W cm^−2^, 5 min). Scale bar = 10 µm. Insets (e), (f), (h), and (i) represent the variations in DCFH‐DA and HPF are assessed via FCM utilizing markers. l) Qualitative analysis of calcein AM and PI probe was used to evaluate the killing effect of MCF7 cells treated with 3ICy5 and 3IZH under 660 nm (10 mW cm^−2^, 5 min) NIR irradiation. Scale bar = 50 µm.

Given the exceptional stability and pH‐controlled release characteristics of 3IZH, this study conducted a series of cell‐based experiments to thoroughly investigate its intracellular mechanisms of action. Initially, the uptake of 3ICy5 and 3IZH by MCF‐7 cells over 3.5 h was analyzed. It is observed that both 3ICy5 and 3IZH exhibited pronounced red fluorescence within this timeframe, indicating effective internalization by the MCF‐7 cells and demonstrating good cellular uptake capabilities (Figure S10, Supporting Information). According to previous reports, the cationic nature of Cy5 imparts targeting ability toward negatively charged organelles, such as the ER or mitochondria.^[^ ^22^ ^]^ The colocalization coefficient of 3ICy5 and 3IZH with the ER was evaluated using an ER green fluorescence probe, yielding a coefficient of 0.9 (**Figure** **3**a–c), indicating that the compounds exhibit excellent targeting capability toward the ER. Subsequently, the generation of ROS in tumor cells was evaluated among different treatment groups by using the DCFH‐DA probe. The results indicated that the 3IZH + NIR group exhibited the strongest green fluorescence in MCF‐7 and 4T1 cells, indicating the highest ROS generation capability (Figure 3d). FCM analysis consistently confirmed this trend (Figure 3e,f). Then, the HPF probe was further employed to assess the ability of 3IZH to convert endogenous H_2_O_2_ into ·OH through Fenton‐like reaction. In MCF‐7 and 4T1 cells treated with 3IZH + NIR, it can be observed a significantly intense green fluorescence, indicating a high conversion efficiency of endogenous H_2_O_2_ to ·OH within the tumor cells (Figure 3g). FCM analysis yielded consistent results (Figure 3h,i). To further validate the generation efficiency of ·OH in cells, the O27 probe was also utilized, which yielded congruent results (Figure 3j).

Additionally, the SOSG probe was also utilized to evaluate the generation of ^1^O_2_. The results demonstrated that MCF7 and 4T1 cells treated with 3IZH + NIR exhibited the most intense green fluorescence, confirming that 3IZH could produce substantial amounts of ^1^O_2_ under NIR light (Figure 3k). Calcein‐AM and propidium iodide (PI) staining were employed to assess cell viability after different treatments. Co‐culturing MCF7 cells with 3IZH and exposing them to NIR irradiation (660 nm, 1 W cm^−2^, 5 min) resulted in a notable increase in red fluorescence, indicating a highly effective cytotoxic effect (Figure 3l). These experiments collectively confirm that, at the cellular level, 3IZH can selectively target the ER and, under NIR irradiation, generate a significant amount of ROS, thereby inducing oxidative stress in tumor cells and promoting the antitumor process.

**Figure 4:**
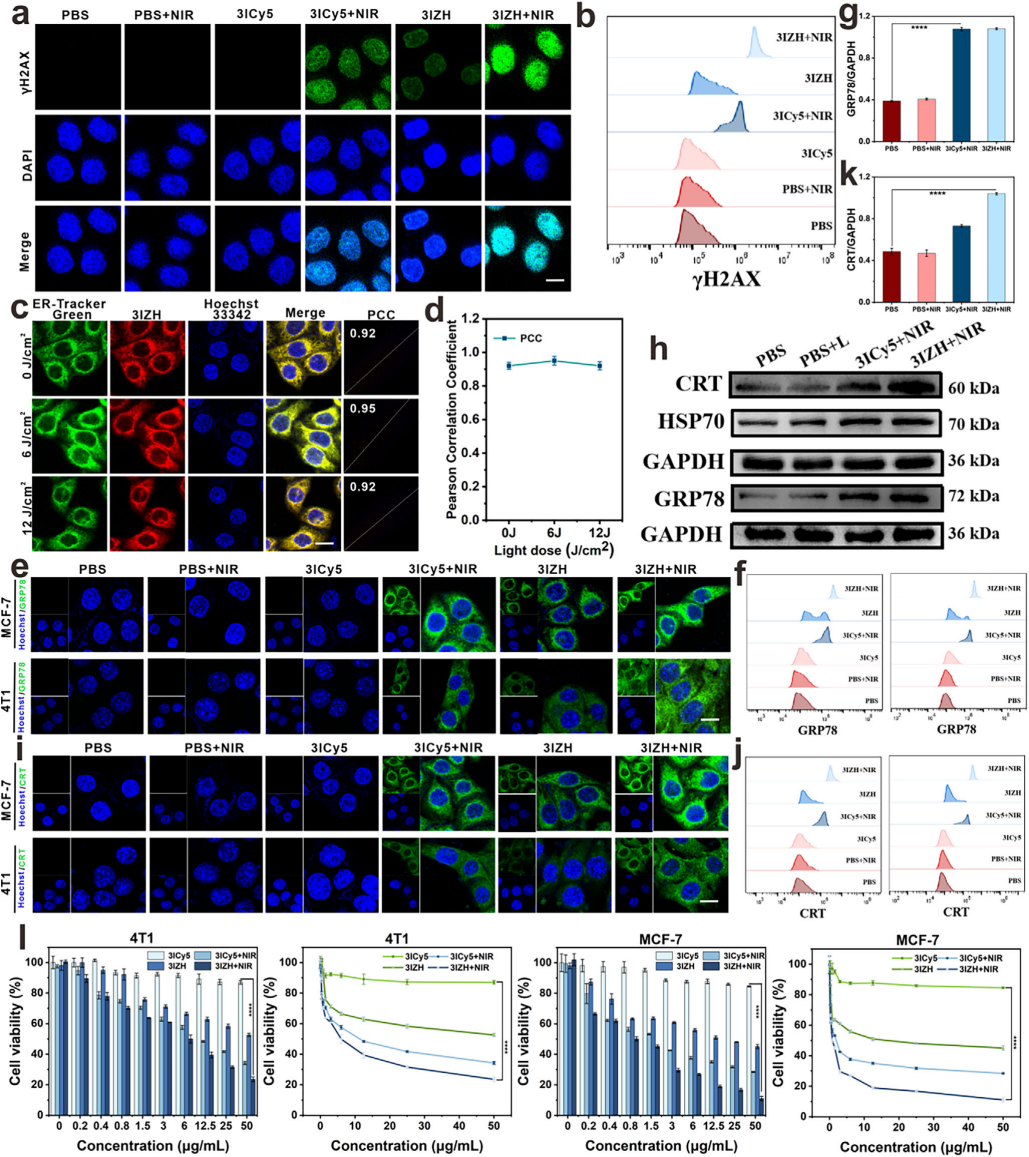
a) The analysis of γH2AX protein expression in MCF7 cells following various treatments using CLSM. Scale bar = 10 µm. b) The quantification of γH2AX protein expression in MCF7 cells following various treatments using flow cytometry. c) Conducting a co‐localization experiment in MCF7 cells with the ER green fluorescent probe and 3IZH. Scale bar = 10 µm. d) Determining the Pearson correlation coefficient between the green and red fluorescent channels. Insets (e) and (i) represent the fluorescence intensities of relevant indicators of cells treated with 3ICy5 or 3IZH after NIR irradiation (660 nm, 1 W cm^−2^, 5 min). Scale bar = 10 µm. Insets (f) and (j) represent the variations in CRT and GRP78 are assessed via FCM utilizing markers. h) Assessment of CRT, HSP70, and GRP78 proteins in 4T1 tumors after different treatments by western blot analysis. g,k) Quantitative analysis of GRP78 and CRT proteins. l) The phototoxicity of 3ICy5 and 3IZH toward 4T1 and MCF7 cells under 660 nm laser irradiation (mean ± SD, n = 3, *p < 0.05, **p < 0.01, and ***p < 0.001). One‐way ANOVA analysis of variance was used for the analysis of statistical significance.

Upon completion of cellular uptake, compound 3IZH, under near‐infrared irradiation, is capable of generating highly efficient ROS. According to widely accepted theories, the substantial release of ROS can lead to DNA damage, subsequently triggering the release of DAMPs.^[^ ^3^, ^23^ ^]^ This mechanism contributes to enhancing the sensitivity of tumor cells to antitumor therapies. In the work, the damage of DNA was analyzed by assessing phosphorylated histone H2AX (γH2AX). MCF7 cells were treated under different conditions. Compared to the other groups, the γH2AX signal intensity in the MCF7 cells treated with 3IZH + NIR showed a significant increase (**Figure** **4**a). This phenomenon validates that after absorption by tumor cells, 3IZH, under NIR irradiation, effectively induces the release of ROS, which in turn triggers the release of DAMPs, leading to DNA damage. As a result, the generation of γH2AX is boosted. FCM was employed to evaluate the expression of γH2AX protein, which also aligned with the observations from laser confocal microscopy (Figure 4b).

The endoplasmic reticulum‐targeting properties of 3IZH induce endoplasmic reticulum stress, subsequently activating the release of calreticulin (CRT), glucose regulatory protein 78 (GRP78), and heat shock protein 70 (HSP70).^[^ ^24^, ^25^ ^]^ This process enhances the functionality of antigen‐presenting cells, thereby stimulating the immune system to adopt an active defence strategy, which effectively suppresses tumor growth and progression. The targeting efficiency of 3IZH under different light intensities was assessed. Notably, under varying NIR radiation conditions, 3IZH consistently demonstrated strong targeting to the ER (Figure 4c,d). Subsequently, the markers associated with ICD were evaluated. The ER stress response resulted in the upregulation of GRP78 expression. After co‐incubation of 3IZH with MCF7 and 4T1 cells, followed by NIR irradiation (10 mW cm^−2^, 5 min), quantitative analysis using a GRP78 green fluorescence probe revealed a significant enhancement in fluorescence intensity in the 3IZH + NIR group (Figure 4e). This result was consistent with the findings obtained from FCM analysis (Figure 4f). Subsequently, protein expression levels of relevant proteins were assessed using Western blot analysis. The experimental results indicated a significant increase in the expression of GRP78 in the 3IZH + NIR treatment group, thereby confirming that 3IZH effectively enhances GRP78 expression by inducing an endoplasmic reticulum stress response (Figure 4g,h). Assessment of CRT expression levels showed that the green fluorescence intensity in the 3IZH + NIR group was significantly higher compared to other groups (Figure 4i), aligning with FCM results (Figure 4j). Similar results were obtained through Western blot analysis, which also demonstrated an up‐regulation in the expression of CRT (Figure 4h,k). The expression level of HSP70 was assessed using a fluorescence probe, and the results revealed the highest expression in the 3IZH + NIR group (Figure S11, Supporting Information). Similar results were obtained through Western blot analysis (Figure 4h). Afterward, cytotoxicity in different treatment groups was further evaluated. Under dark conditions, the cell viability of cells treated with 50 µg mL^−1^ 3ICy5 exceeded 80%, indicating minimal cytotoxicity. After NIR irradiation (660 nm, 10 mW cm^−2^, 5 min), the viability of 4T1 cells decreased to 34.3% (3ICy5) and 23.5% (3IZH). The calculated half‐maximal inhibitory concentration (IC_50_) demonstrated that the IC_50_ of 3IZH (1.2 µg mL^−1^) was significantly lower than that of 3ICy5 (1.8 µg mL^−1^) (Figure 4l), indicating the synergistic effect between the introduced ions and PSs. Given that the upregulation of CRT, GRP78, and HSP70 is a characteristic marker of DAMPs, the results of this study demonstrate that, under NIR irradiation conditions, 3IZH effectively induces ICD in tumor cells to modulate the typically immunosuppressive “cold” microenvironment.

### 2.3 Induction of Ferroptosis by 3IZH and the Impact of HIF‐1α Deletion on Ferroptosis Resistance

Ferroptosis is a distinct form of cell death characterized by iron‐dependent lipid peroxidation within the cell, leading to the oxidation of polyunsaturated fatty acids on the cell membrane.^[^ ^26^, ^27^, ^28^, ^29^ ^]^ This process results in the disruption of membrane integrity and ultimately triggers cell death. 3IZH induces the release of Fe^3^⁺, oxidizes GSH, and inhibits GPX4, leading to the accumulation of LPO within the tumor cells, thereby triggering ferroptosis.

**Figure 5:**
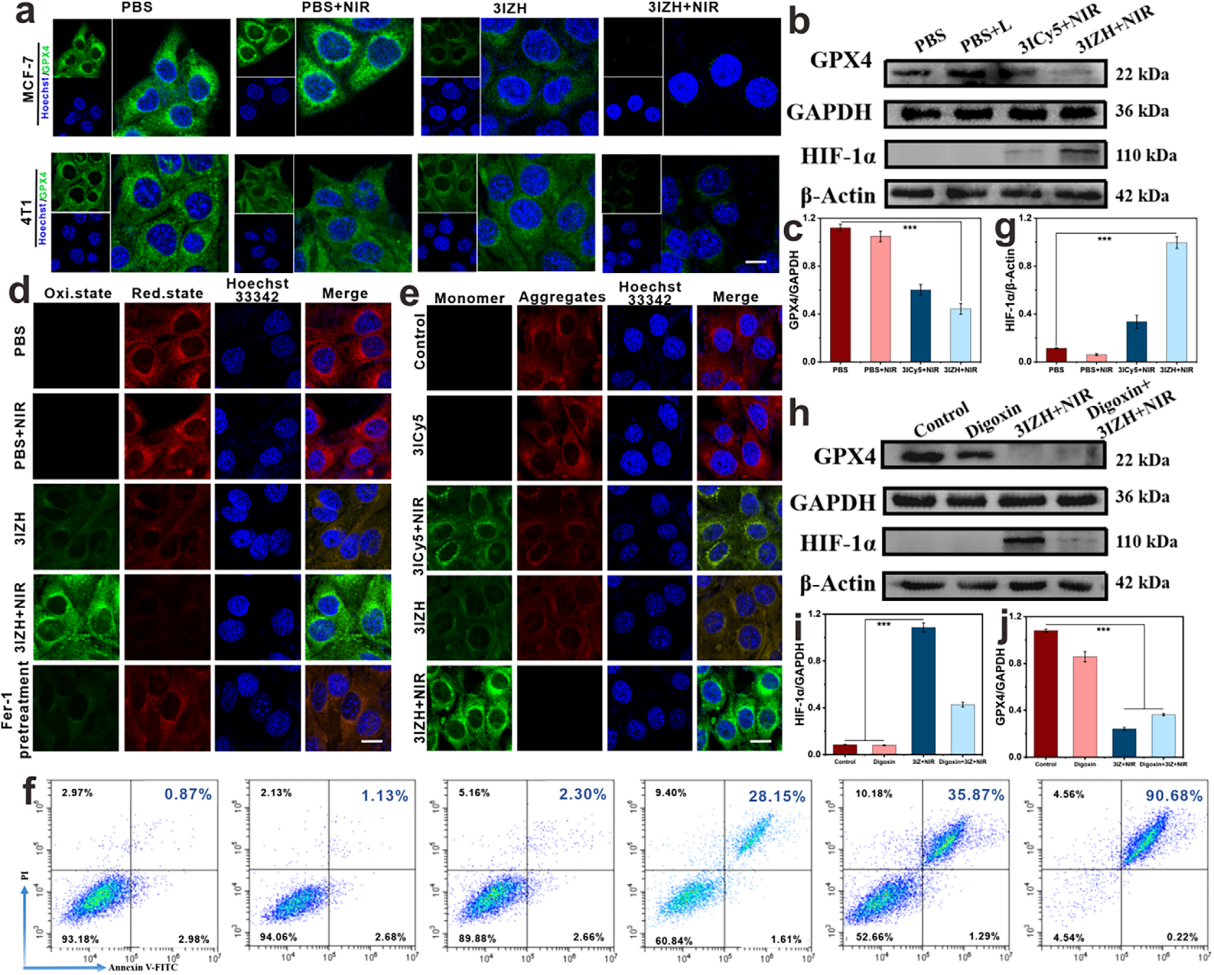
a) Fluorescence staining of GPX4 in MCF7 and 4T1 cells is treated by 3IZH. Scale bar = 10 µm. b,h) Assessment of GPX4 and HIF‐1α proteins in 4T1 tumors after different treatments by western blot analysis. c,g,i,j) Quantitative analysis of GPX4 and HIF‐1α proteins. d) Detection of lipid peroxidation was performed in 4T1 cells treated with 3IZH. Scale bar = 10 µm. e) Detection of Mitochondrial membrane potential was performed in 4T1 cells treated with 3ICy5 and 3IZH. Scale bar = 10 µm. f) FCM analysis of MCF7 cells treated with Annexin V‐FITC and PI (mean ± SD, n = 3, *p < 0.05, **p < 0.01, and ***p < 0.001). One‐way ANOVA analysis of variance was used for analysis of statistical significance.

To elucidate the underlying mechanisms of 3IZH‐induced ferroptosis, the expression levels of relevant proteins were first assessed by using western blotting. The results demonstrated a significant decrease in GPX4 expression levels in the 3IZH treatment group, confirming that 3IZH effectively inhibits the GPX4 expression (**Figure** **5**a–c). To further investigate the mechanism of action, the researchers utilized the C11‐BODIPY probe to assess the levels of LPO in 4T1 cells treated with 3IZH. The experimental data revealed that under oxidative conditions, the 3IZH‐treated group exhibited the most intense green fluorescence signal, which was almost completely quenched following pre‐treatment with Fer‐1. These findings suggest that 3IZH induces ferroptosis by promoting the accumulation of LPO, thereby compromising membrane integrity and leading to tumor cell death (Figure 5d). Additionally, it was observed that the mitochondrial membrane potential is significantly reduced in the 3IZH + NIR group (Figure 5e), which further supports that 3IZH effectively induced ferroptosis in tumor cells via LPO accumulation and suppression of GPX4 expression. Moreover, the cell apoptosis was quantified by using FCM. The results revealed that the apoptosis rate in the 3IZH + NIR group significantly increased to 90.68% compared to the PBS group (Figure 5f).

Hypoxia, as a prevalent feature of the TME, has been shown in multiple studies to promote the resistance mechanisms of tumor cells to ferroptosis and apoptosis.^[^ ^30^, ^31^, ^32^ ^]^ Specifically, the upregulation of HIF‐1α may undermine the efficacy of therapeutic strategies that rely on inducing ferroptosis and apoptosis in tumor cells to achieve sustained anti‐tumor effects. To validate this hypothesis, the present study employed western blotting to assess the expression levels of HIF‐1α. The experimental results revealed that under ferroptosis stress conditions, the expression of HIF‐1α in the 3IZH + NIR treatment group showed a significant upregulation (Figure 5b,g). This suggests that the up‐regulation of HIF‐1α may be attributed to the exacerbated hypoxic state induced by oxygen depletion during photodynamic therapy, or it could result from a rapid adaptive response mechanism triggered by ferroptotic stress. However, prolonged activation of the HIF‐1α stress response not only induces resistance to ferroptosis and apoptosis in tumor cells but also promotes tumor angiogenesis, invasion, and metastasis, accelerating tumor progression.^[^ ^33^, ^34^ ^]^

To address this challenge, our study further employed digoxin, an HIF‐1α inhibitor, in combination with 3IZH. A cell scratch assay was first performed under simulated hypoxic conditions in HepG2 and 4T1 cells to establish a model of sustained HIF‐1α activation. Subsequent scratch experiments revealed that persistent HIF‐1α activation significantly promoted cell migration in both HepG2 and 4T1 cells. However, combined treatment with digoxin and 3IZH effectively suppressed this HIF‐1α mediated enhancement of cell migration (Figure S12, Supporting Information). These results demonstrate that HIF‐1α is a key regulator of cell migration, and that our therapeutic strategy based on digoxin and 3IZH can effectively block its function.^[^ ^35^ ^]^ We further evaluated whether this combination treatment could attenuate the up‐regulation of HIF‐1α under stress conditions using Western blot analysis. The experimental results demonstrated that HIF‐1α expression was significantly reduced in the digoxin + 3IZH+NIR combined treatment group compared to the 3IZH+NIR group (Figure 5h,i). This finding indicates that the combined application of digoxin and 3IZH nanoparticles effectively inhibits the expression of HIF‐1α, thereby holding promise for reversing the resistance of tumor cells to ferroptosis and apoptosis. Further observation revealed that the combination of Digoxin and 3IZH nanoparticles led to a sustained decrease in GPX4 expression (Figure 5h,j), which further confirmed that this combined treatment significantly enhances the sensitivity of tumor cells to ferroptosis. Taken together, these results demonstrate that the co‐application of 3IZH nanoparticles and Digoxin holds potential advantages in overcoming hypoxia‐induced ferroptosis and apoptosis resistance, providing a novel perspective for the application of nanoparticles in long‐term therapeutic strategies for solid tumors.

### 2.4 NIR‐Mediated Combination of 3IZH and Digoxin Enhances Anti‐Tumor Immunity In Vitro and In Vivo

In the field of tumor immunotherapy, inducing the polarization of M2 macrophages to M1 macrophages is considered one of the key strategies to overcome the immunosuppressive state within the TME.^[^ ^36^, ^37^, ^38^ ^]^ This study investigates the potential of combining 3IZH and Digoxin under NIR irradiation to induce ICD, a process believed to enhance immune responses and promote macrophage polarization. To assess the efficacy of the 3IZH and Digoxin combination therapy in stimulating anti‐tumor immune responses, we evaluated its immunological effects in 4T1 tumor‐bearing mice.

**Figure 6:**
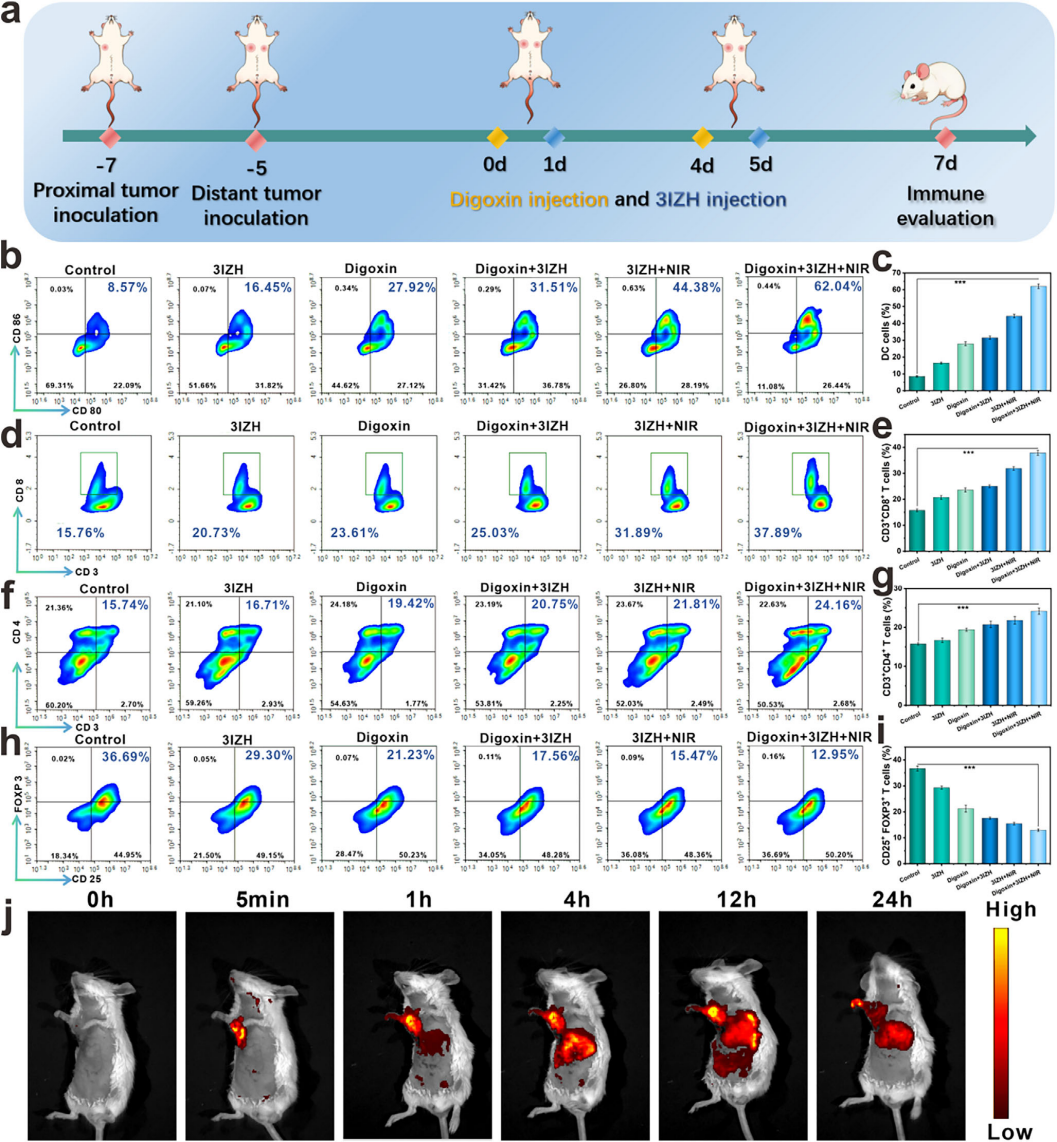
a) Model construction and research process. Insets (b), (d), (f), and (h) represent the expression of DC cells, CD3^+^ CD8^+^ T cells, CD3^+^ CD4^+^, Treg cells T cells detected by using FCM methodology. Insets (c), (e), (g), and (i) respectively represent the quantitative analysis of DC cells, CD3^+^ CD8^+^ T cells, CD3^+^ CD4^+^, Treg cells T cells. j) Time‐dependent fluorescence imaging of subcutaneous 4T1 tumor‐bearing mice after i.v. injection of 3IZH (mean ± SD, n = 3, and *p < 0.05, **p < 0.01, and ***p < 0.001). One‐way ANOVA analysis of variance was used for the analysis of statistical significance.

As shown in **Figure** **6**a, the bilateral 4T1 tumor model was established in mice, with proximal and distant tumors designed to correspond to day 7 and day 5 before treatment initiation, respectively. Mice received Digoxin on days 0 and 4. On days 1 and 5, 3IZH treatment was administered, followed by 660 nm laser irradiation (10 mW cm^−2^, 5 min) of the primary tumor 4 h post‐treatment. Tumor growth was monitored every other day following treatment. Subsequently, FCM was used to analyze the status of immune‐related cells. Enhancing DC maturation can effectively amplify T cell activation and subsequent immune response. Memory T cells play a crucial role in driving and regulating immune responses against tumor‐associated antigens, and they are also essential in maintaining long‐term anti‐tumor immunity. First, we validated the ability of the 3IZH and Digoxin combination to activate immune responses by monitoring the maturation of DCs and T cell infiltration. Compared to the PBS group, the DCs maturation rate in the Digoxin + 3IZH + NIR group increased from 8.57% to 62.04% (Figure 6b,c). A similar trend was observed in the distant tumors (Figure S13, Supporting Information). The proportions of CTLs were then evaluated. In the Digoxin + 3IZH + NIR group, the proportion of CD3^+^ CD8^+^ T cells reached 37.89%, while it was only 15.76% in the PBS group (Figure 6d,e). A similar trend was noted in the distant tumors (Figure S14, Supporting Information). The proportion of CD3^+^ CD4^+^ T cells increased from 15.74% in the PBS group to 24.16% (Figure 6f,g), with corresponding trends observed in the distant tumors (Figure S15, Supporting Information). Regulatory T cells, known for their immune‐suppressive functions, exhibited a significant reduction in the proportion within the primary tumors of the Digoxin + 3IZH + NIR group compared to the PBS group (Figure 6h,i). A similar trend was also found in the distant tumors (Figure S16, Supporting Information). To further assess the exhaustion status of CD8⁺ T cells, we measured the expression levels of exhaustion markers PD‐1 and LAG‐3 on the surface of CD8⁺ T cells. As shown in Figure S17 (Supporting Information), compared to the control group, the expression of PD‐1 and LAG‐3 proteins in the tumor tissues of the treatment group was significantly reduced. The substantial decrease in the total protein levels of these mature exhaustion markers strongly indicates that our therapeutic intervention can effectively improve the exhaustion state of CD8⁺ T cells in vivo.

To evaluate the in vivo biodistribution and near‐infrared fluorescence imaging capability of 3IZH nanoparticles, the researchers employed the IVIS Spectrum imaging system to track tumor‐associated fluorescence signals in real time. As shown in Figure 6j, significant accumulation of 3IZH in the tumor region was observed 4 hours post‐injection, followed by a gradual decrease. After 24 hours of injection, ex vivo semi‐quantitative imaging analysis of the heart, liver, spleen, lungs, kidneys, and tumor tissues revealed that 3IZH predominantly accumulated in the tumor tissue (Figure S18, Supporting Information). These results collectively affirm the significant therapeutic efficacy of the Digoxin‐3IZH combination with NIR treatment in suppressing both primary and distant tumor growth. This indicates that the strategy may enhance anti‐tumor effects by modulating immune responses within the TME, thereby inducing ICD, providing new insights and methodologies for tumor immunotherapy.

### 2.5 Treatment of Bilateral Tumor Models

**Figure 7:**
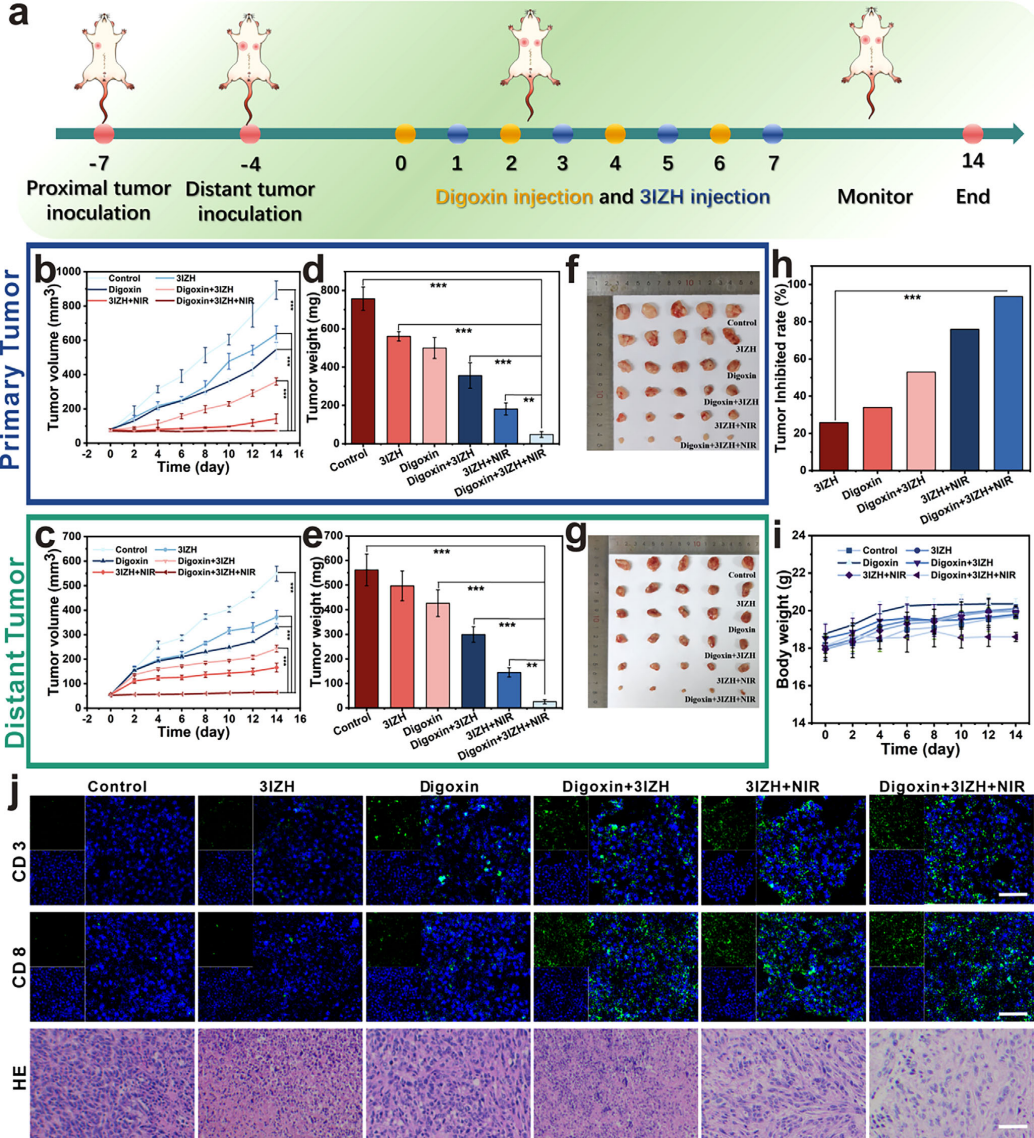
a) Mouse model generation process diagram. b) The volume of proximal tumors in mice (n = 5). c) The volume of distal tumors in mice (n = 5). d) The weight of proximal tumors in mice (n = 5). e) The weight of distal tumors in mice (n = 5). f) Images of primary tumors in mice (n = 5). g) Images of distal tumors in mice (n = 5). h) Inhibitory rates of tumor after different treatments (n = 5). i) Body weight of mice during the experiment (n = 5). j) Tumor tissues were stained with H&E, CD3 molecule and CD8 molecule after 14 days of different treatments. (n = 3). Scale bar = 50 µm. (mean ± SD, n = 3, and *p < 0.05, **p < 0.01, and ***p < 0.001). One‐way ANOVA was used to assess significance.

To further elucidate the synergistic mechanism of the Digoxin‐3IZH combination therapy, we assessed its potentiating effects in a bilateral 4T1 tumor‐bearing mouse model. To ensure that the drug acted effectively on the tumors, 3IZH was applied by direct intratumor injection. Mice were randomly divided into six groups (n = 5): Control group, 3IZH group, Digoxin group, Digoxin + 3IZH group, 3IZH+NIR group, and Digoxin + 3IZH + NIR group. The tumor volumes of each group were recorded every two days. The therapeutic scheme is summarized in **Figure** **7**a. After the end of the treatment, it was clear that the tumors of the control mice injected with the control grew most rapidly. The combined strategy of Digoxin and 3IZH proposed in this study aims to enhance the dual anti‐tumor effects of PDT and ferroptosis, demonstrating a significant tumor‐suppressive effect. The results of this study not only confirm the effectiveness of the combination therapy we developed in enhancing tumor cell cytotoxicity, but also provide a novel research avenue for advancing the efficacy of anti‐tumor treatments (Figure 7b–h). There were no significant changes in the average body weight of mice across all groups, indicating that the compounds possessed high safety and low toxicity (Figure 7i). Histological examination of organs, including heart, liver, spleen, lung, and kidney, through H&E staining revealed no significant organ damage in the Digoxin + 3IZH + NIR group, but there was evident damage to tumor tissue (Figure 7j; Figure S19, Supporting Information).

The infiltration of CD3^+^ T cells and CD8^+^ T cells in distant tumors was further assessed by using immunofluorescence staining to confirm the systemic immune activity. Observations revealed a lack of immune responses in the Control group, while the Digoxin + 3IZH + NIR group displayed obvious fluorescence intensity (Figure 7j), indicating significant infiltration of CD3^+^ T cells and CD8^+^ T cells into tumor cells, which could stimulate a systemic immune response and effectively transition the TME into an immunologically activated state.

## 3 Conclusion

This study addresses the challenge of achieving effective antitumor immunotherapy by proposing a multifunctional photodynamic‐based immune stimulator. The multifunctional photodynamic immune‐stimulator capable of controllably releasing the 3ICy5 and Fe^3+^ ions in the acidic TME. The ER stress was induced by 3ICy5, further boosting the ROS generation and the DAMPs release, which eventually activated the ICD pathway and activated immunological cells. While the enhanced intracellular Fe pools by supplementing Fe^3+^ from 3IZH induce ferroptosis. To further overcome the ferroptosis‐resistant HIF‐1α, digoxin was further adopted in the combined treatment system. Ultimately, the immunosuppressive TME was successfully reconstructed, significantly enhancing immunogenicity. In vivo experiments demonstrated that 3IZH effectively activated the ICD and ferroptosis pathways for enough immunological cytotoxicity. It significantly suppressed the growth of tumors in the bilateral tumor‐bearing mouse models. It is anticipated that this multifunctional immune stimulator design strategy, which simultaneously activates ICD and ferroptosis pathways, will provide new design concepts and research directions for effective tumor immunotherapy.

## 4 Experimental Section

### Materials

5,5′‐Dithiobis (2‐nitrobenzoic acid) (DTNB, 98%) and 1,3‐diphenylisobenzofuran (DPBF, 95%) were purchased from Bide Pharmatech Ltd (Shanghai, China). Iron chloride (FeCl_3_, ≥ 98%), 2‐methylimidazole (≥ 98%), Rose Bengal (RB, ≥ 90%), 9,10‐anthracenediyl‐bis (methylene) dimalonic acid (ABDA, ≥ 90%), methanol (≥ 99.8%), and dichloromethane (CH_2_Cl_2_, ≥ 99.5%) were acquired from Shanghai Aladdin Biochemical Technology Co., Ltd. ER‐Tracker Green, Hoechst 33342, Mitochondrial Membrane Potential Assay Kit with JC‐1, Annexin V‐FITC Apoptosis Detection Kit, and Cytotoxicity Assay Kit were purchased from Beyotime Biotechnology Co., Ltd. PE Anti‐Mouse CD86 Antibody [GL‐1], FITC Anti‐Mouse CD80 Antibody [16‐10A1], FITC Anti‐Mouse CD3 Antibody [17A2], PE Anti‐Mouse CD8 Antibody [53‐6.7], PE Anti‐Mouse CD4 Antibody [RM4‐5], FITC Anti‐Mouse CD25 Antibody [PC‐61.5.3], and PE Anti‐Mouse Foxp3 Antibody [3G3] were obtained from Proteintech Biotechnology Co., Ltd. 3‐(4,5‐dimethyl‐2‐thiazolyl)‐2,5‐diphenyl‐2‐Htetrazolium bromide (MTT, ≥ 97%) was purchased from Energy Chemical Co., Ltd. Hydroxyphenyl fluorescein (HPF, ≥ 95%) was purchased from Shanghai Maokang Biotechnology Co., Ltd. Calcein‐AM/PI Detection Kit, GSH Assay Kit, GPX4 Rabbit Polyclonal Antibody, were purchased from Beyotime Biotechnology Co., Ltd. Mouse breast cancer cells (4T1) and Michigan Cancer Foundation‐7 (MCF‐7) were purchased from the Institute of Basic Medical Sciences (IBMS) of the Chinese Academy of Medical Sciences.

### Methods

^1^H‐NMR and ^13^C‐NMR spectra were measured using a Bruker Avance II 400 spectrometer. Mass spectrometric (ESI‐MS) data were detected utilizing an Ultimate 3000 (Thermo Scientific) instrument. Fluorescence images were captured using a TCS SP8 confocal laser scanning microscope (Leica). Cell apoptosis detection was conducted via FCM (BECKMAN COULTER), and the cytotoxicity experiment was carried out using a multifunctional microporous reader (SpectraMax i3x). The morphological structures of 3IZH were observed by the TEM (FEI Company, USA). DLS and zeta potential measurements were conducted with a ZS nanohybrid analyzer (Malvern, England). UV–vis–NIR absorption spectra of different samples were recorded by an Evolution 220 UV–vis spectrophotometer (UV‐2700i, Japan). The values of Fe in different valence states were analyzed using XPS (ESCA Lab 250, Thermo Fisher Scientific, USA) experiments.

### Synthesis of Compound 1

In a three‐necked round‐bottom flask, 2,3,3‐trimethyl‐3H‐indole (2.14 g, 13.47 mmol) and 1‐(bromomethyl)‐4‐iodobenzene (4.01 g, 13.47 mmol) were weighed and dissolved in 5 mL of acetonitrile. The mixture was thoroughly stirred and then heated in an oil bath for 12 hours at the set temperature. Upon completion of the reaction, the mixture was allowed to stand for a period of time to ensure complete separation of the product from the solvent system. Finally, the resulting pale‐yellow solid was collected by vacuum filtration. The solid was identified as compound 1 (3.74 g, 73.7% yield).

### Synthesis of Compound 2

POCl_3_ (2 mL) was added dropwise to 9 mL of dimethylformamide (DMF) under ice bath conditions and allowed to react for 1 hour. The reaction mixture was then stirred at room temperature for an additional 3 hours. Subsequently, 1.50 grams of 2‐(4‐iodophenyl) acetic acid was introduced, and the mixture was heated to 90 °C, followed by reduced‐pressure distillation and reflux for 6 hours. Upon completion of the reaction, the solution was cooled to room temperature, and crushed ice was added. Under stirring, an excess of sodium perchlorate (NaClO_4_) was introduced, leading to the formation of a significant amount of light‐yellow solid. The solid was filtered and washed twice with a supersaturated sodium perchlorate solution to remove impurities, yielding the intermediate product. Without further purification, the intermediate was transferred directly into 20 mL of sodium hydroxide solution (0.80 g, 10 mmol) and heated under reflux at 90 °C until the light yellow solid completely dissolved. The solution was then cooled to room temperature, and 10 mL of deionized water was added to dilute the mixture. Finally, the pH of the solution was adjusted to 2 using 20% hydrochloric acid, and the precipitate was isolated by vacuum filtration, yielding compound 2 as a light‐yellow solid (1.44 g, 91.8% yield).

### Synthesis of 3ICy5

Compound 1 (2.0 g, 5.31 mmol) and compound 2 (0.73 g, 2.68 mmol) were combined in a 50 mL three‐necked round‐bottom flask and thoroughly mixed. To this mixture, 7 mL of anhydrous ethanol and three drops of pyridine were added. The reaction mixture was then heated to 80 °C and allowed to react under reflux conditions with continuous condensation for 10 hours. The reaction progress was monitored by thin‐layer chromatography (TLC). Following the reduction of the yellow intermediate, the solvent was removed under reduced pressure. The final product, 3ICy5 (1.89 g, 73.5% yield), was purified by column chromatography using a dichloromethane/methanol mixture (20:1 v/v) as the elution solvent, yielding the desired compound. ^1^H NMR (400 MHz, MeOD) δ 8.23 (d, J = 14.1 Hz, 2H), 7.72 (d, J = 7.7 Hz, 4H), 7.64 (d, J = 8.1 Hz, 6H), 7.55 (d, J = 7.5 Hz, 2H), 7.45 – 7.38 (m, 2H), 7.36 (d, J = 7.8 Hz, 2H), 7.30 (t, J = 7.4 Hz, 2H), 6.64 (d, J = 7.9 Hz, 4H), 4.99 (s, 4H), 1.76 (d, J = 2.1 Hz, 12H). ^13^C NMR (101 MHz, MeOD) δ 173.72, 152.78, 142.61, 141.10, 138.36, 138.03, 134.25, 131.36, 128.69, 127.91, 125.47, 122.41, 110.51, 103.16, 92.61, 67.88, 49.14, 38.71, 31.65, 30.46, 29.31, 29.03, 28.71, 26.49, 22.64, 22.33, 13.12, 13.08. HRMS (ESI): m/z calc. for [C_45_H_40_I_3_N_2_]^+^ 989.03, found 989.03 [M]^+^.

### Synthesis of 3IZ

Dissolve a total of 20 mg of 3ICy5 in 5 mL of ethanol to obtain a homogeneous solution. Under continuous stirring, an ethanol solution containing 4 mg of ZnFe‐ZIF was slowly added to the 3ICy5 solution. The mixture was stirred for 1 hour to ensure thorough mixing. The mixture solution was kept in the dark and stirred overnight at room temperature. Then, the solvent was removed under reduced pressure using a rotary evaporator until the residue was fully dry. Finally, the resulting residue was reconstituted in PBS buffer to a concentration of 200 µg mL^−1^ to obtain the desired solution.

### Synthesis of 3IZH

For the preparation of 3IZH, 15 mg of HA was added to the 3IZH solution and stirred overnight for 12 h. Following this, centrifugation at 10 000 rpm for 12 min was performed, and sample 3IZH was washed twice with distilled water.

### Generation of Singlet Oxygen by 3IZH

The generation capability of ^1^O_2_ by 3IZH was evaluated using DPBF as a probe. Specifically, 3IZH (10 µg mL^−1^) was dispersed in 5 mL of DPBF (50 µM) aqueous solution. The mixture was irradiated with a 660 nm laser at a power density of 1 W cm^−2^ for 10 min. Subsequently, the generation of ^1^O_2_ was assessed using UV–vis spectroscopy. ABDA and SOSG were also employed to detect ^1^O_2_, with the experimental process similar to that of DPBF, only substituting the probe with ABDA or SOSG. Subsequently, the fluorescence emission spectra of SOSG and the UV–vis absorption spectra of ABDA were recorded within 5 min, respectively.

### General Physicochemical Characterization

The morphology of the nanoparticles was characterized using a Tecnai G2 20 S‐TWIN TEM operating at 200 kV. Size distribution and zeta potential were measured with a DLS analyzer, specifically the Zeta sizer Nano ZS90 (Malvern Instruments Ltd). XRD patterns were obtained using a powder X‐ray diffraction instrument (Rigaku Smartlab), with measurements conducted using Cu Kα radiation over a 2θ range of 15–50^°^ and the scanning step speed is 5^°^/min. XPS measurements were performed using a Thermo Scientific K‐Alpha spectrometer (USA) with Al Kα radiation as the X‐ray source. The survey scan was conducted in the binding energy range of 0–1200 eV, with a pass energy of 150 eV, and high‐resolution scans were carried out for specific elements with a pass energy of 30 eV.

### Determination of Fe^3+^

The determination of the Fe^3+^ release from 3IZH was conducted using an atomic absorption spectrometer. Initially, 5 mg of the material was incubated separately in 10 mL of PBS at pH 7.4, 6.5, and 5.3, respectively. At various time points, 100 µL of the samples were collected and diluted 40 times before quantitatively analyzing the Fe^3+^ content.

### Cell Culture

MCF7, 4T1, and HepG2 cells were cultured in DMEM medium supplemented with 10% fetal bovine serum and 1% antibiotic solution (penicillin/streptomycin, 100 U mL^−1^). The cells were maintained at a constant temperature of 37 °C in a 5% CO_2_ and 95% air atmosphere.

### Cytotoxicity Assay

4T1 cells were seeded in a 96‐well plate at a density of 1 × 10^4^ cells per well, with 100 µL of medium added to each well, and incubated at 37 °C for 24 h. After the removal of the original medium, varying concentrations (0–100 µg mL^−1^) of 3ICy5 and 3IZH solutions were introduced, and the cells were further incubated in a temperature‐controlled chamber for an additional 2 h. Subsequently, the samples were subjected to 660 nm light irradiation (1 W cm^−2^ for 5 min). Following the light exposure, the cells were allowed to incubate for another 24 h. Subsequently, 20 µL of MTT solution (5 mg mL^−1^) was added to each well, and the cells were incubated for an additional 4 h. The prior medium was then discarded, and 150 µL of dimethyl sulfoxide was added to each well, followed by gentle mixing for 10 min. The absorbance at 570 nm was measured using a microplate reader (Spectra Max i3x). Cell viability was calculated using the following Equation (1):

(1) $$\mathrm{Cell}\;\mathrm{Viability}\;\left(\%\right)=\frac{OD_{\mathrm{PS}}-OD_{\mathrm{black}\;\mathrm{control}}}{OD_{\mathrm{control}}-OD_{\mathrm{black}\;\mathrm{control}}}\times 100\%$$

In addition, the cytotoxicity of 3ICy5 and 3IZH toward MCF7 cells was assessed using the same MTT assay. Cells were incubated with serially diluted concentrations of each drug for 24 h, and the dose‐dependent inhibitory effects were analyzed by comparing cell viability across the concentration gradients.

### Cell Uptake Test

The cellular internalization of 3ICy5 and 3IZH was evaluated at multiple time intervals. Cells were seeded into laser confocal culture dishes with 1 mL of medium, achieving a cell density of 1 × 10^5^ per milliliter, and were incubated in a controlled‐temperature environment for 24 h. Subsequently, 3ICy5 was added at a concentration of 1.8 µg mL^−1^ at various time points, and 3IZH was added at a concentration of 1.2 µg mL^−1^ at various time points. Following the treatment, the cells underwent three washes with PBS and were subsequently examined using a CLSM to visualize the internalization process.

### Western Blot Analysis of 4T1 Cells Treated with 3IZH

At the onset of the experiment, 4T1 cells were seeded in 6 cm culture dishes and cultured with a medium containing 3IZH at a concentration of 5.1 µg mL^−1^. Following a 2‐hour incubation in the dark, the cells were exposed to 660 nm light for 5 min. The incubation continued for an additional 6 h, after which the cells were detached using 0.25% trypsin and washed three times with PBS. Subsequently, the cells were lysed using a cell lysis buffer supplemented with protease and phosphatase inhibitors and maintained on ice for 30 min. The protein concentration was determined using the Bradford assay, and equal amounts of protein were loaded into each lane of an SDS‐PAGE gel for electrophoresis. Following electrophoresis, the proteins were transferred to a polyvinylidene difluoride (PVDF) membrane. The membranes were blocked with 5% BSA and incubated overnight at 4 °C with a primary antibody against GPX4. Afterward, a horseradish peroxidase (HRP)‐ conjugated goat anti‐rabbit secondary antibody was applied and incubated at room temperature for 1 h. Finally, the immunoblots were developed using an ECL detection system.

Western Blot Analysis of CRT, GRP78, HSP70, HIF‐1α, PD‐1, and LAG‐3 were measured using the same methodology, with the sole distinction of substituting the GPX4 primary antibody with the CRT, GRP78, HSP70, HIF‐1α, PD‐1, and LAG‐3 primary antibodies.

### Detection of Intracellular ^1^O_2_

Single cells were plated in laser confocal culture dishes at a density of 1 × 10^5^ cells per mL. The dishes were then incubated in a cell culture incubator for 24 h until the cells adhered completely. After plating, the dishes were divided into six experimental groups: PBS group, PBS + NIR group, 3ICy5 group, 3ICy5 + NIR group, 3IZH group, and 3IZH + NIR group. Following a 1‐hour incubation period in the culture incubator, the green fluorescent probe DCFH‐DA (10 µM) was introduced, and the cells were allowed to incubate for an additional hour. The PBS + NIR, 3ICy5 + NIR, and 3IZH + NIR groups were subjected to near‐infrared light irradiation (660 nm, 10 mW cm^−2^) for 5 min. After light exposure, the old medium was discarded, and the cells were washed three times with PBS. Subsequently, CLSM was employed for observation.

### Detection of Intracellular Hydroxyl Radicals (•OH)

The detection of •OH was performed using the HPF probe, following the same procedural steps as those employed with the DCFH‐DA probe, with the sole modification being the replacement of DCFH‐DA with the HPF probe.

### Subcellular Localization of Cells

Cells were dispensed into laser confocal culture dishes and incubated at 37 °C for 24 h. Following this, a fresh culture medium containing 3IZH (1.2 µg mL^−1^) was added, and the cells were further incubated for an additional 2 h. At the end of the incubation period, subcellular localization probes were introduced to the laser confocal culture dishes as per the manufacturer's instructions, followed by another 1‐hour incubation. The cells were then washed three times with PBS, after which fresh culture medium was added, and observations were made using a CLSM.

### Cell Viability Assessment

Initially, cells were extracted from 25 T culture flasks and subsequently plated into laser confocal culture dishes to achieve a density of 1 × 10^5^ cells per milliliter. These dishes were placed in a cell incubator and incubated for 24 h to ensure complete adhesion of the cells to the surface. According to the experimental protocol, the culture dishes were divided into six groups: PBS, PBS + NIR, 3ICy5, 3ICy5 + NIR, 3IZH, and 3IZH + NIR. The cells were allowed to incubate in the cell culture incubator for an additional 6 h. Thereafter, the PBS + NIR, 3ICy5 + NIR, and 3IZH + NIR groups were exposed to NIR light at a wavelength of 660 nm and an intensity of 10 mW cm^−2^ for 5 min. Postirradiation, the cells were incubated for an additional 6 h in the cell culture incubator before the appropriate reagents from the cell viability assay kit were added. The cells were then incubated at a constant temperature for 2 h, followed by three washes with PBS. Finally, the dishes were examined under CLSM.

### Apoptosis Detection

Cells extracted from 25 T culture flasks were seeded into 6‐well plates at a density of 1 × 10^5^ cells per milliliter. The plates were subsequently placed in a cell incubator for 24 h until the cells adhered completely. Following the experimental design, the cells were allocated into six groups: PBS, PBS + NIR, 3ICy5, 3ICy5 + NIR, 3IZH, and 3IZH + NIR. As required, 3ICy5 (1.8 µg mL^−1^) and 3IZH (1.2 µg mL^−1^) were added, and the cells were incubated for an additional 2 h in the cell culture incubator. Subsequently, the PBS + NIR, 3ICy5 + NIR, and 3IZH+ NIR groups were subjected to NIR light exposure at 660 nm and an intensity of 10 mW cm^−2^ for 5 min. Following light exposure, the cells were incubated for an additional 12 h in the cell culture incubator. After enzymatic digestion, appropriate detection reagents were added, and the samples were subjected to machine detection.

### Wound Healing Assay

HepG2 or 4T1 cells were seeded at a density of 2 × 10⁶ cells per well in a 6‐well plate and incubated in complete medium for 24 hours. After reaching confluence, a sterile pipette tip was used to create a uniform scratch across the cell monolayer. Following two washes with PBS, the cells were incubated in low‐serum medium (≤ 2%) containing either Digoxin + 3IZH (at the indicated experimental concentrations) or DMSO (as a vehicle control). The cells were initially incubated for 1 hour under normoxic conditions, then transferred to a hypoxic environment for an additional 24 hours. At 0 and 24 hours post‐scratch, typical images were captured using an inverted microscope. The scratch area (S) was measured using ImageJ software. Wound healing assay was calculated using the following Equation (2).

(2) $$\mathrm{Migration}\mathrm{Index}=\frac{S_{0h}-S_{24h}}{S_{0h}}$$

### Expression of GRP78 Protein

Cells were cultured at a density of 1 × 10^6^ cells per milliliter in laser confocal culture dishes. Subsequently, the dishes were placed in an incubator for 24 h to allow for complete cell adhesion to the surface. Based on the experimental requirements, the cells were divided into six groups: PBS group, PBS + NIR group, 3ICy5 group, 3ICy5 + NIR group, 3IZH group, and 3IZH + NIR group. After treatment with 3ICy5 and 3IZH, the cells were further incubated in the incubator for an additional 2 h, followed by exposure to NIR light (660 nm, 1 W cm^−2^) for 5 min. Following illumination, the old culture medium was aspirated, and the cells were fixed overnight using an immunostaining fixative. After fixation, the cells were incubated with a primary antibody against GRP78 for 4 h. Subsequently, the primary antibody was removed, and the dishes were washed with PBS before incubation with a secondary fluorescent antibody for an additional 4 h. After this incubation, the secondary antibody was removed, and the dishes were washed with PBS. The samples were then observed under CLSM.

### Expression of HSP70 Protein

Following the same protocol as described above, the only modification involved substituting the GRP78 primary antibody with the HSP70 primary antibody.

### Expression of CRT Protein

The methodology was consistent with the aforementioned protocol, with the sole distinction of substituting the GRP78 primary antibody with the CRT primary antibody.

### Expression of the GPX4 Protein

Cells from the 25 T culture flask were individually cultured in laser confocal culture dishes at a density of 1 × 10^6^ cells per milliliter. Subsequently, the dishes were placed in a cell culture incubator and incubated for 24 h until the cells fully adhered to the surface. Following the experimental requirements, the cells were divided into six groups: PBS group, PBS + NIR group, 3ICy5 group, 3ICy5 + NIR group, 3IZH group, and 3IZH + NIR group. The cells were further incubated for an additional 2 h and then exposed to near‐infrared light (660 nm, 1 W cm^−2^) for 5 min. Following light exposure, the old culture medium was aspirated, and the cells were fixed overnight with an immunostaining fixative solution. After fixation, the cells were incubated with a GPX4 primary antibody for 4 h. Subsequently, the primary antibody was removed, and the culture dishes were washed with PBS before continuing incubation for another 4 h with a fluorescent secondary antibody. After this incubation, the secondary antibody was removed, and the dishes were washed with PBS. Finally, the samples were observed under a laser confocal microscope.

### Animals

Female BALB/c mice (6–8 weeks old) were purchased from provided by Beijing Vital River Laboratory Animal Technology Co., Ltd. (Beijing, China) and maintained in a specific pathogen‐free (SPF) environment. All animal studies were conducted in compliance with the guidelines established by the Animal Experiment Ethics Review Board of Shandong Second Medical University (Approval number 2022SDL381). Initially, the mice were allowed to acclimatize to their new environment for one week. For tumor implantation, 1 × 10^6^ 4T1 cells suspended in 100 µL PBS were injected either subcutaneously into the left axillary region. Upon reaching the third day of left‐side tumor growth, tumors were implanted on the right side using the same method.

### Anti‐Tumor Efficacy Study

BALB/c mice bearing subcutaneous 4T1 tumors (≈100–200 mm^3^) were randomly assigned to six treatment groups: Control group, 3IZH group, Digoxin group, Digoxin + 3IZH group, 3IZH + NIR group, and Digoxin + 3IZH + NIR group. The treatments targeted the left tumor, the mice received intratumoral injections of 3IZH (0.4 mg kg^−1^) and Digoxin. Four hours post‐injection, the mice were subjected to NIR irradiation (660 nm, 20 mW cm^−2^). Tumor growth was monitored regularly to evaluate the residual tumor response. Tumor volumes and tumor inhibition rates were calculated using the following formulas, Equations (3) and (4):

(3) $$\mathrm{Tumor}\;\mathrm{volume}\;\left(\mathrm{Vt},\;mm^{3}\right)=\frac{a\;\times b^{2}}{2}$$

where a was the major axis and b was the minor axis of the tumor.

(4) $$\mathrm{Tumor}\;\mathrm{inhibition}\;\mathrm{rate}\;\left(\%\right)=\frac{V_{c}-V_{t}}{V_{c}}\times 100\%$$

Here, V_c_ was the mean tumor volume (or weight) of the control group, and V_t_ was that of the treatment group at the study endpoint.

### Detection of Surface Molecular Expression in Mouse Tumors

Following the extraction of murine tumor cells, the tumor tissues were meticulously minced using surgical scissors. The minced tissues were then subjected to a 30‐minute digestion period with collagenase at 37 °C. After digestion, the cell suspension was resuspended in PBS and filtered through a 300‐mesh filter membrane. The resulting cell suspension was centrifuged at 1500 rpm for 5 min. The pellet was subsequently resuspended in buffer, with 600 µL of buffer added to each tube. Thereafter, 30 µL aliquots were taken for staining, which was performed for 1 h before analysis using the instrument.

### Statistical Analysis

The data were presented as mean ± standard deviation (S.D.) with the indicated sample size in figure legends. For multiple groups, one‐way single factorial ANOVA was carried out to ascertain the statistical difference of the data. GraphPad Prism 9.5.1 and Microsoft Excel 2021 were used for data statistics and statistical significance calculation. *P* < 0.05 was taken as statistically significant (*P < 0.05, **P < 0.01, and ***P < 0.001).

## Conflict of Interest

The authors declare no conflict of interest.

## Supporting information



Supporting Information

## Data Availability

The data that support the findings of this study are available from the corresponding author upon reasonable request.
