# Supplementary material for: Multifunctional Nano Immunostimulant: Overcoming Immunosuppressive Microenvironment for Antitumor Immunotherapy
Source: Adv Sci (Weinh). 2025 Dec 12;13(12):e17480. doi: 10.1002/advs.202517480 (PMC12948280; doi:10.1002/advs.202517480)
Supplement: Supplementary file 1 — Supporting Information [file ADVS-13-e17480-s001.docx]

**Supporting information**

**Multifunctional Nano Immunostimulant: Overcoming** **Immunosuppressive Microenvironment for Antitumor Immunotherapy**

*Guanhong Guo^1,#^, Wenda Zhong^1,#,^*, Huishuang Zhao^1,#^, Yueying An^1^, Xinyu Dong^1^, Zhengbo Li^1^, Shuangfeng Qin^1^, Guangzhao Xu^1^, Xiangguo Yue^2^, Xudong Wang^3^, Wen Sun^4^, Zhe-Sheng Chen^5^, Weiguo Song^1,^*, Liuya Wei^1,^*, Fahui Li^1,^**

*1 G. H. Guo, W. D. Zhong, H. S. Zhao, Y. Y. An, X. Y. Dong, Z. B. Li, S. F. Qin, G. Z. Xu, Prof. W. G. Song, Prof. L. Y. Wei, Prof. F. H. Li.*

*School of Pharmacy, Shandong Second Medical University, Weifang 261053, People’s Republic of China*

*2 X. G. Yue*

*Weifang University of Science and Technology, Weifang 262700, People’s Republic of China*

*3 X. D. Wang*

*Harway Pharma (Weifang) Co., Ltd. Weifang 262700, People’s Republic of China*

*4 Prof. W. Sun*

*State Key Laboratory of Fine Chemicals, Dalian University of Technology, Dalian 116024, China*

*5* *Prof. Z.-S. Chen*

*Department of Pharmaceutical Sciences, College of Pharmacy and Health Sciences, St. John’s University, New York, NY, United States*

*^#^These authors contributed equally to this work.*

**Corresponding authors.*

*E-mail: zhongwd@sdsmu.edu.cn; songwg@sdsmu.edu.cn; weily@sdsmu.edu.cn; lifh@sdsmu.edu.cn.*

**Scheme S1.** The synthesis route for **3ICy5**


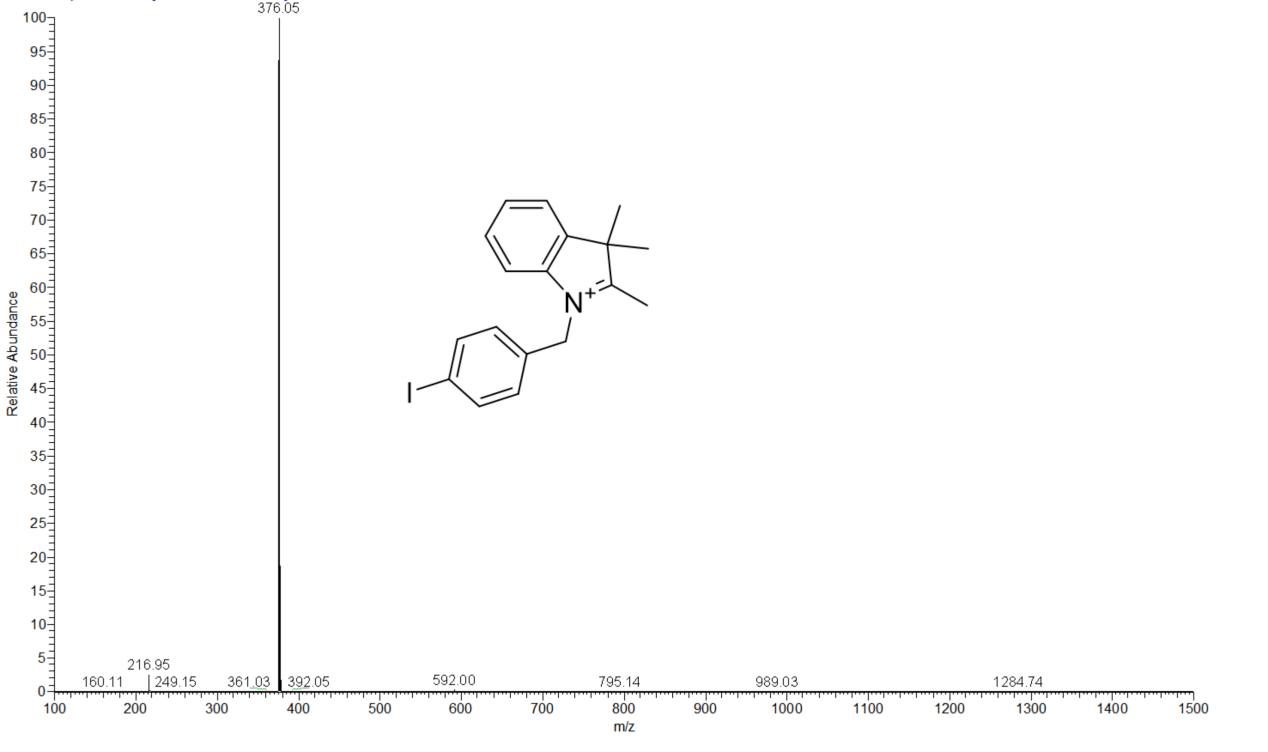


**Figure S1.** Mass spectrum of compound 1


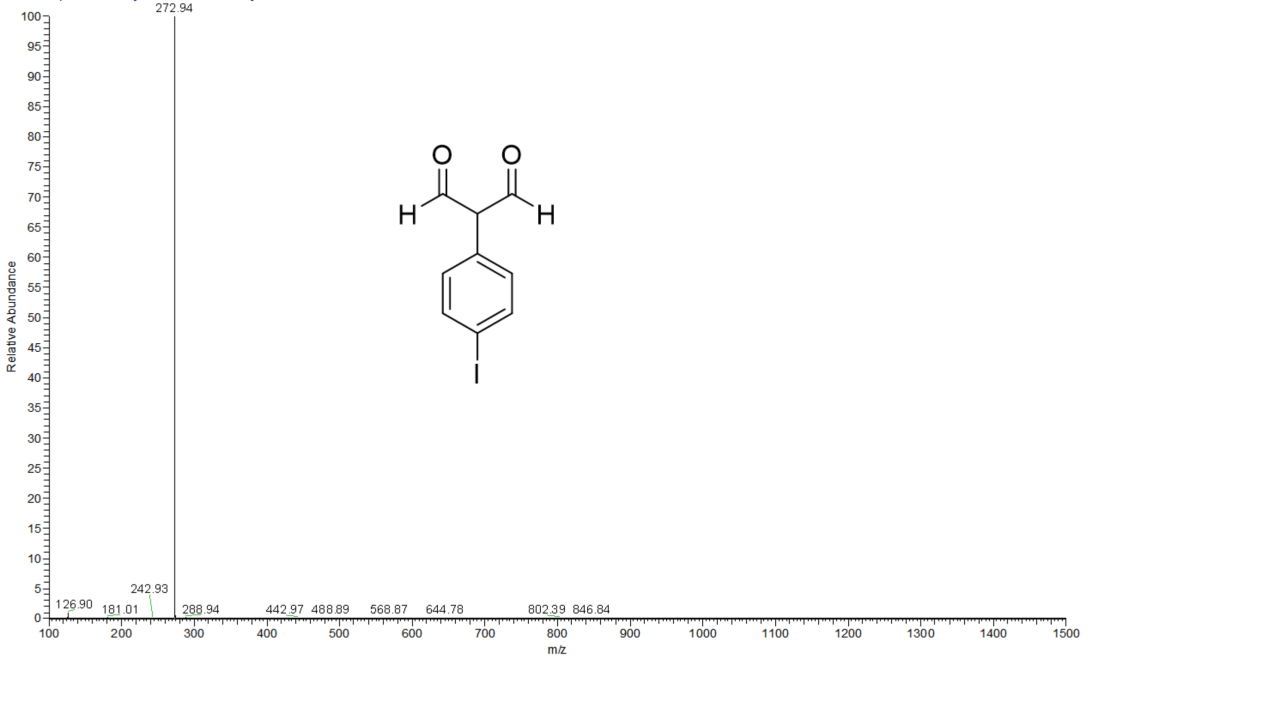


**Figure S2.** Mass spectrum of compound 2


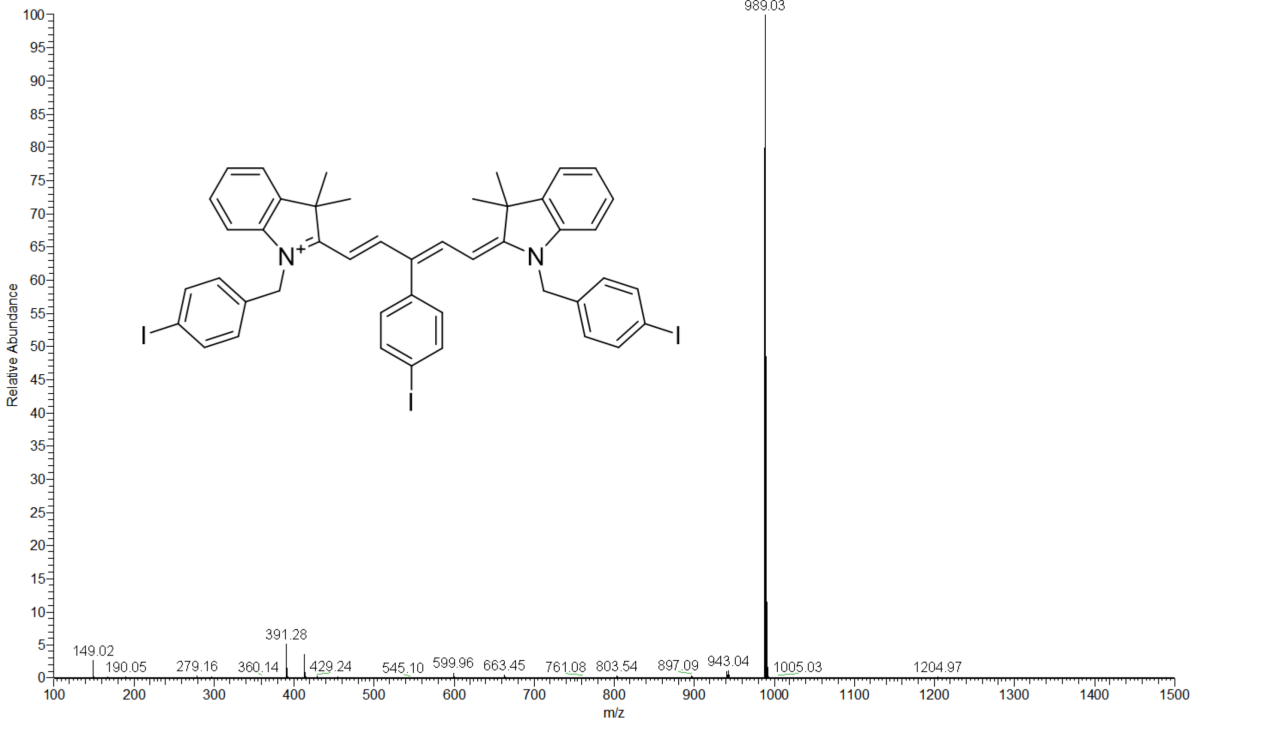


**Figure S3.** Mass spectrum of 3ICy5


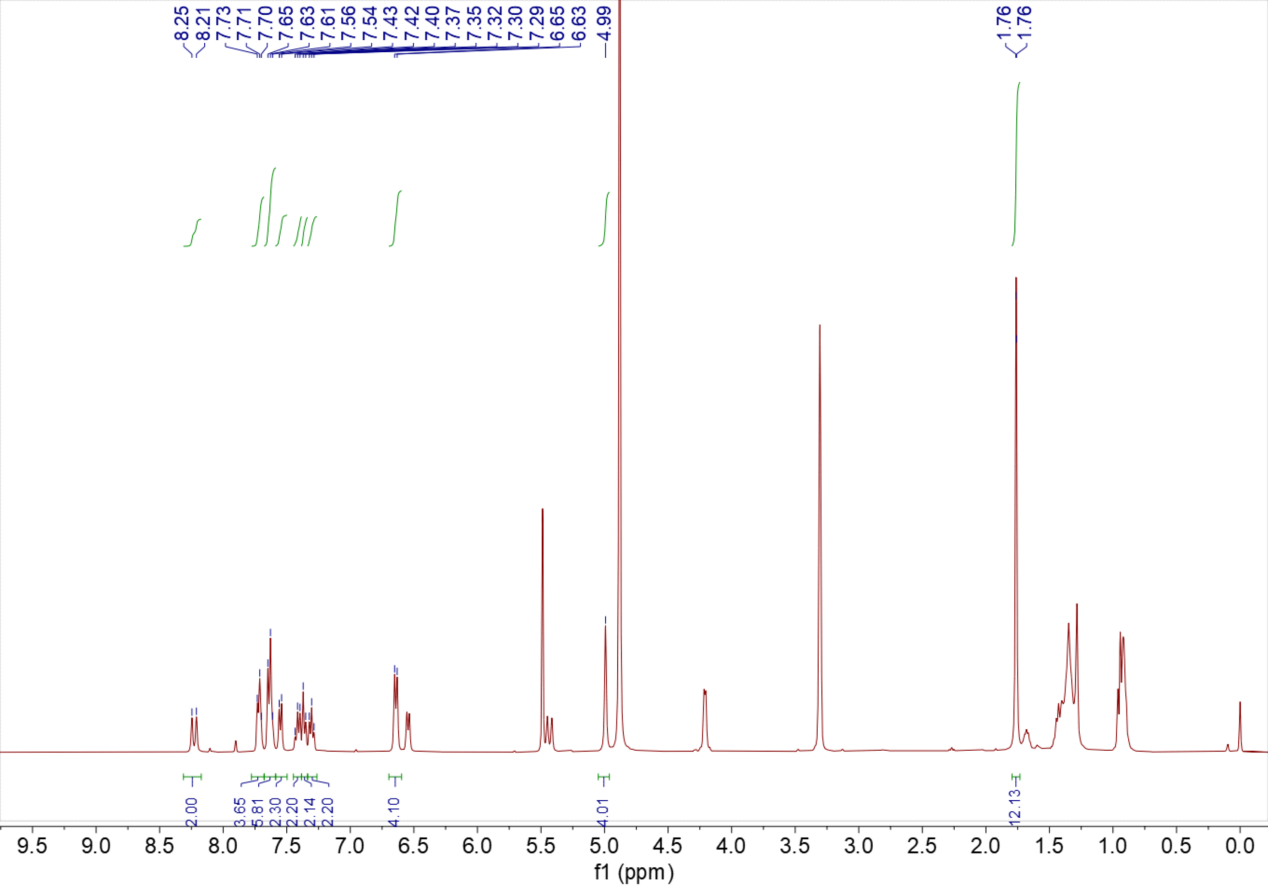


**Figure S4.** ^1^H NMR spectrum of 3ICy5 (400 MHz, MeOD).


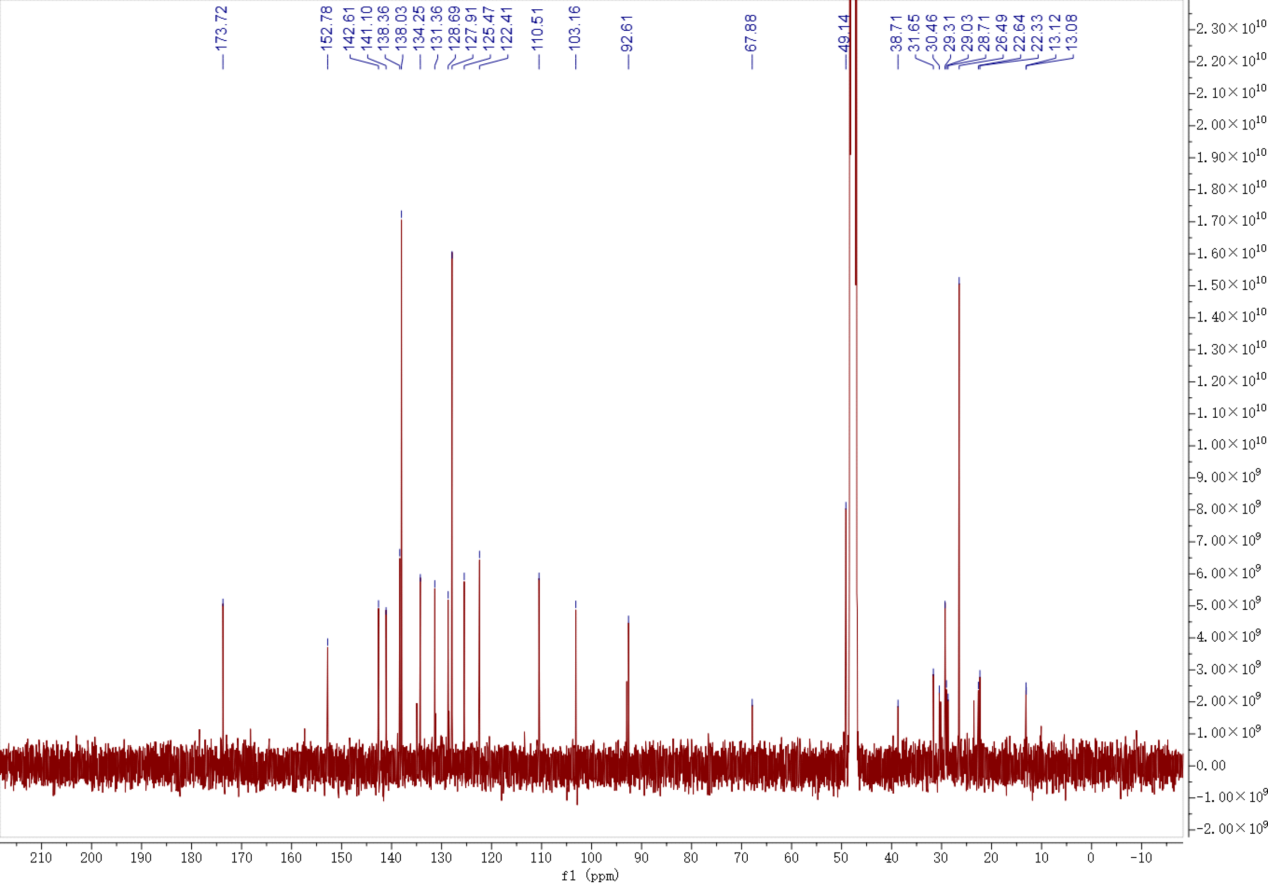


**Figure S5.** ^13^C NMR spectrum of 3ICy5 (101 MHz, MeOD).


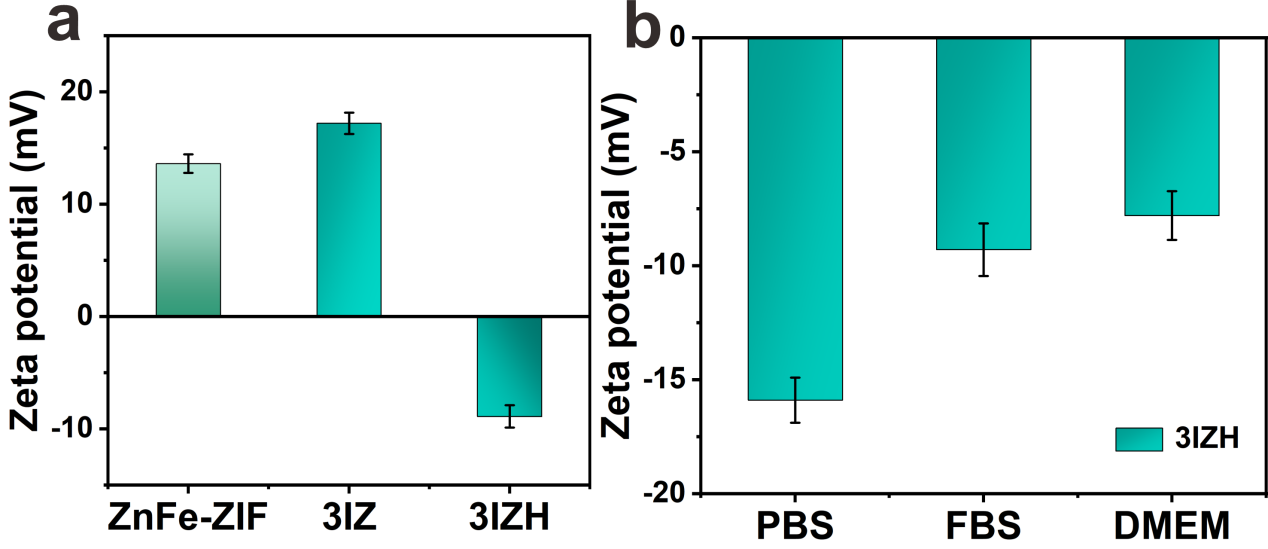


**Figure S6.** (a) Zeta potential of ZnFe-ZIF, 3IZ, and 3IZH. (b) Zeta potential of 3IZH in PBS, FBS and DMEM solution.


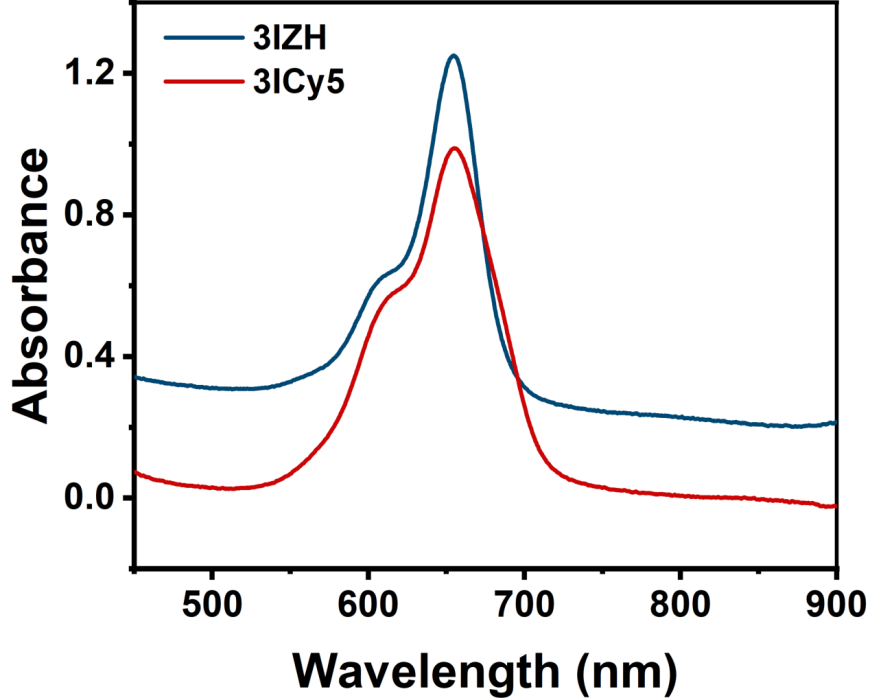


**Figure S7.** UV-Vis absorption of 3ICy5 and 3IZH in PBS (PH = 6.5).


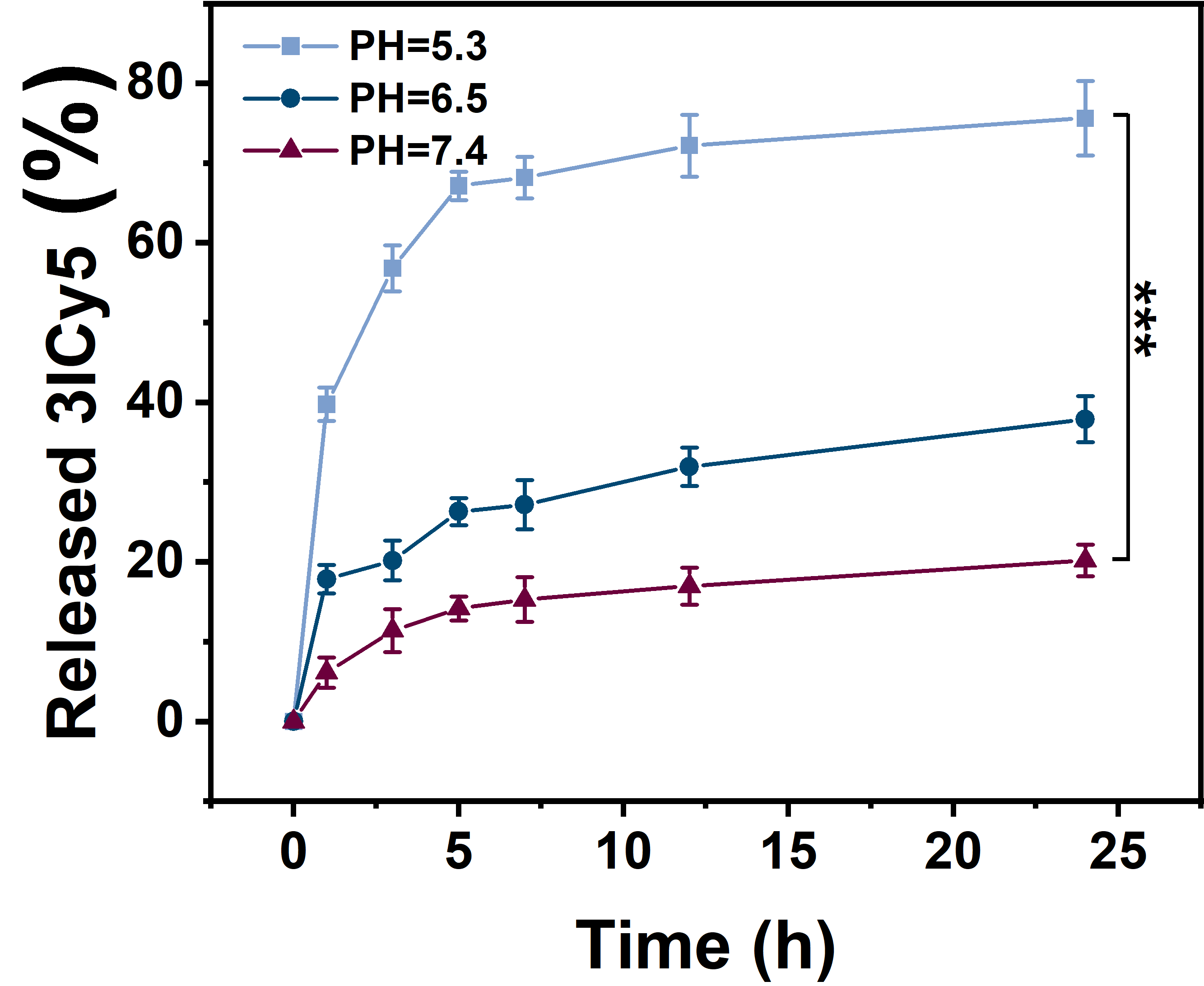


**Figure S8.** Fe-released profile of 3IZH at different pH values (mean ± SD, n = 3, *p < 0.05, **p < 0.01 and ***p < 0.001). One-way ANOVA was used to assess significance.


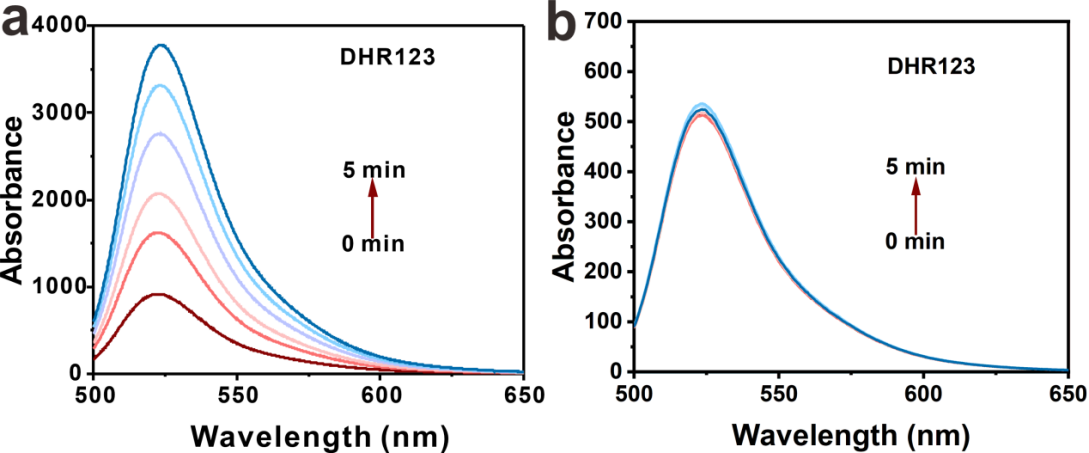


**Figure S9.** (a) ROS generation under NIR light irradiation (660 nm, 10 mW/cm^2^) after co-incubation of DHR123 with 3IZH. (b) ROS generation by DHR123 under NIR light irradiation (660 nm, 10 mW/cm^2^) alone.


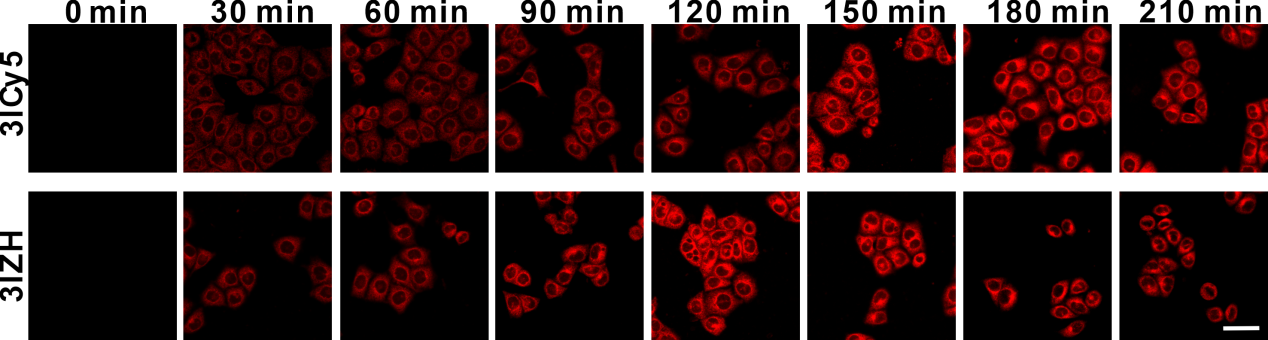


**Figure S10.** Cellular uptake of 3ICy5 and 3IZH in MCF-7 cells. Emissions were collected at 750-800 nm (λ_ex_ = 660 nm) during the experiment; Scale bar = 20 μm.


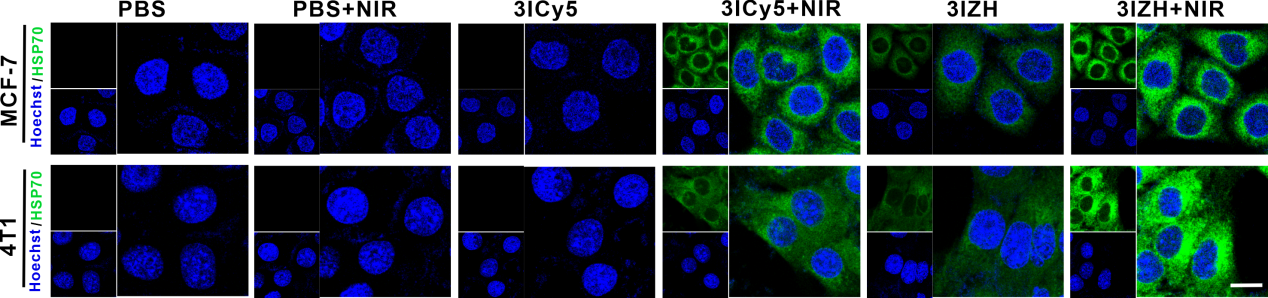


**Figure S11.** The protein fluorescence of HSP70 was measured using fluorescence detection. For the HSP70, emissions were collected at 500-600 nm (λ_ex_ = 490 nm); Scale bar = 10 μm.


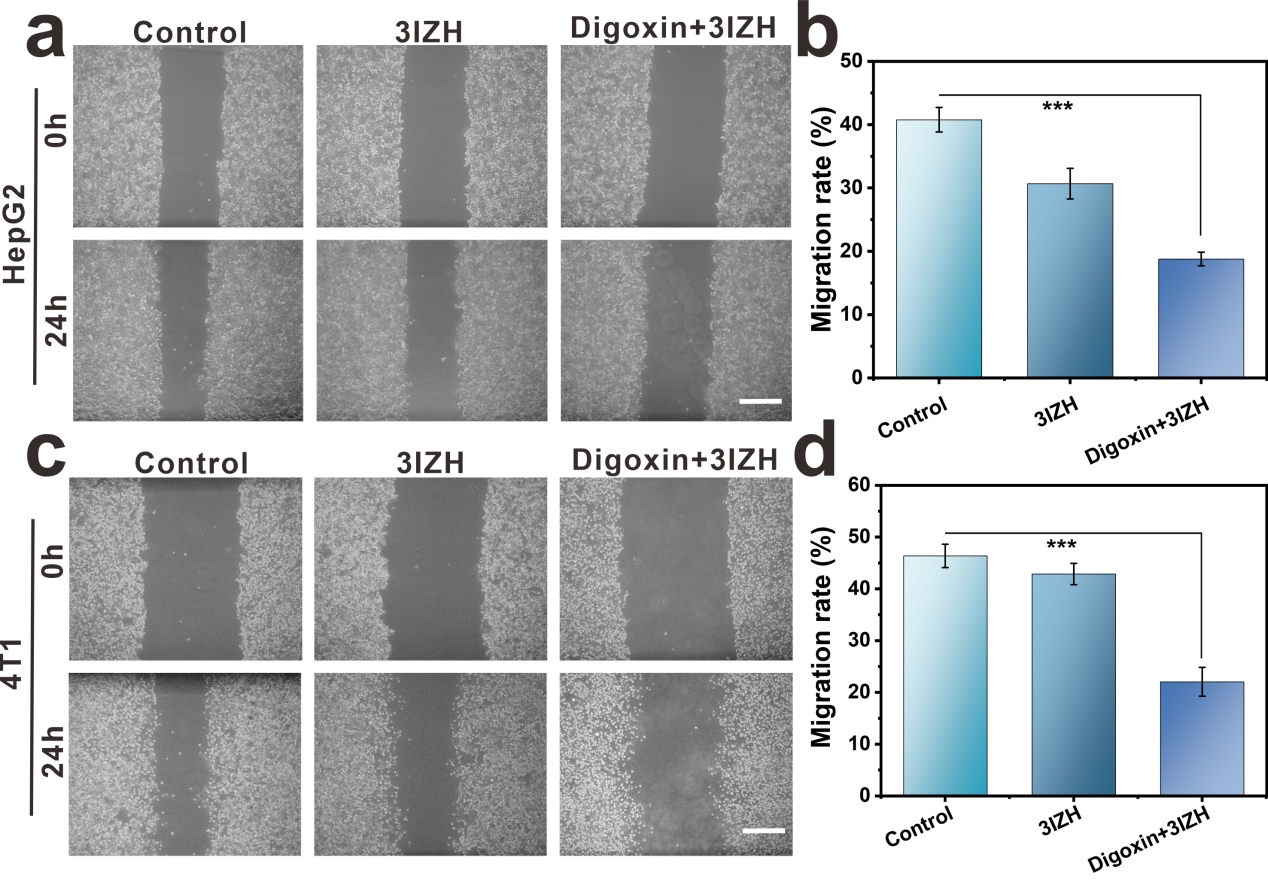


**Figure S12.** Wound healing images of HepG2 and 4T1 cells after treatment; Scale bar = 100 μm. (mean ± SD, n = 3, and *p < 0.05, **p < 0.01 and ***p < 0.001). One-way ANOVA was used to assess significance.


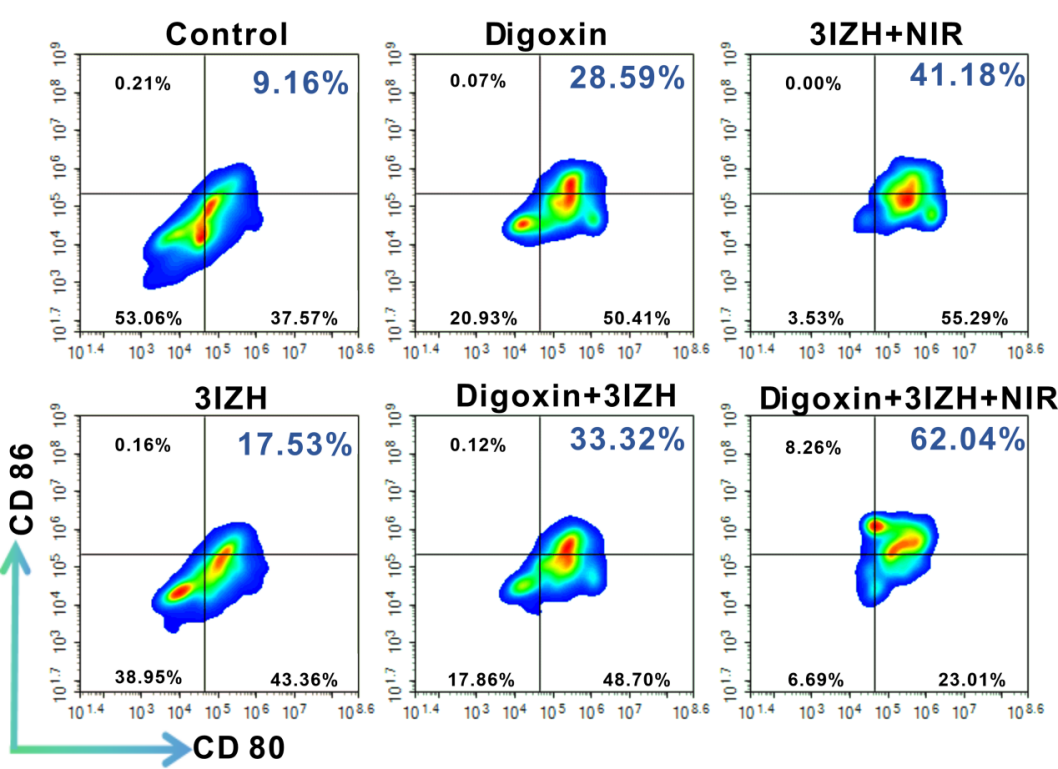


**Figure S13.** Flow cytometric detection of CD80^+^ CD86^+^ on the surface of mouse tumors (distant tumors). (mean ± SD, n = 3).


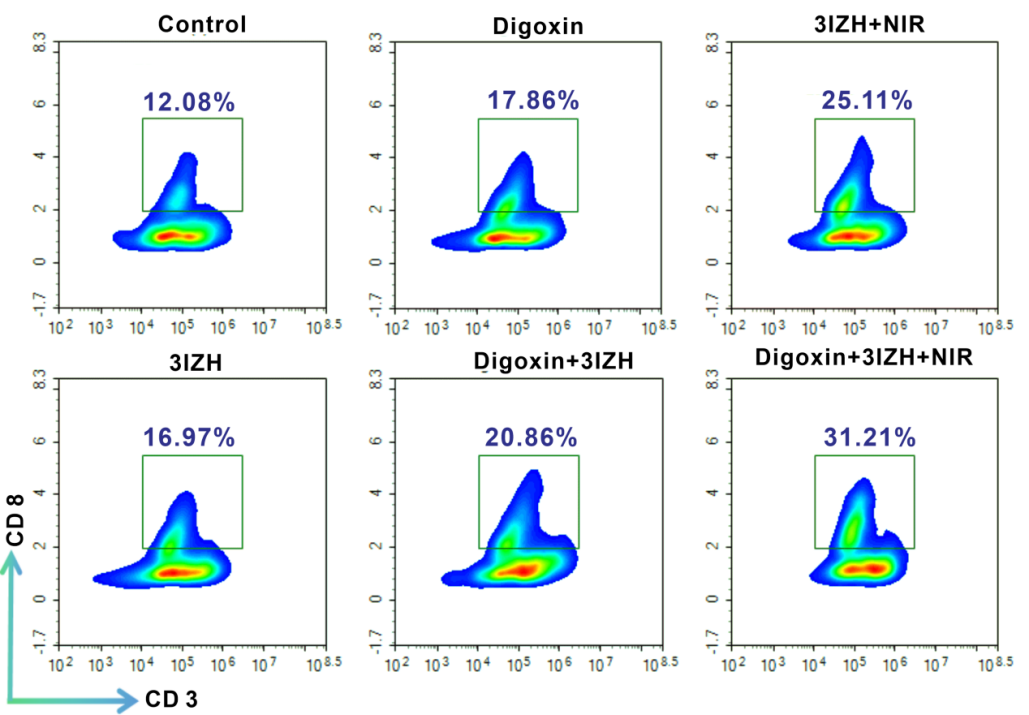


**Figure S14.** Flow cytometric detection of CD3^+^ CD8^+^ on the surface of mouse tumors (distant tumors). (mean ± SD, n = 3).


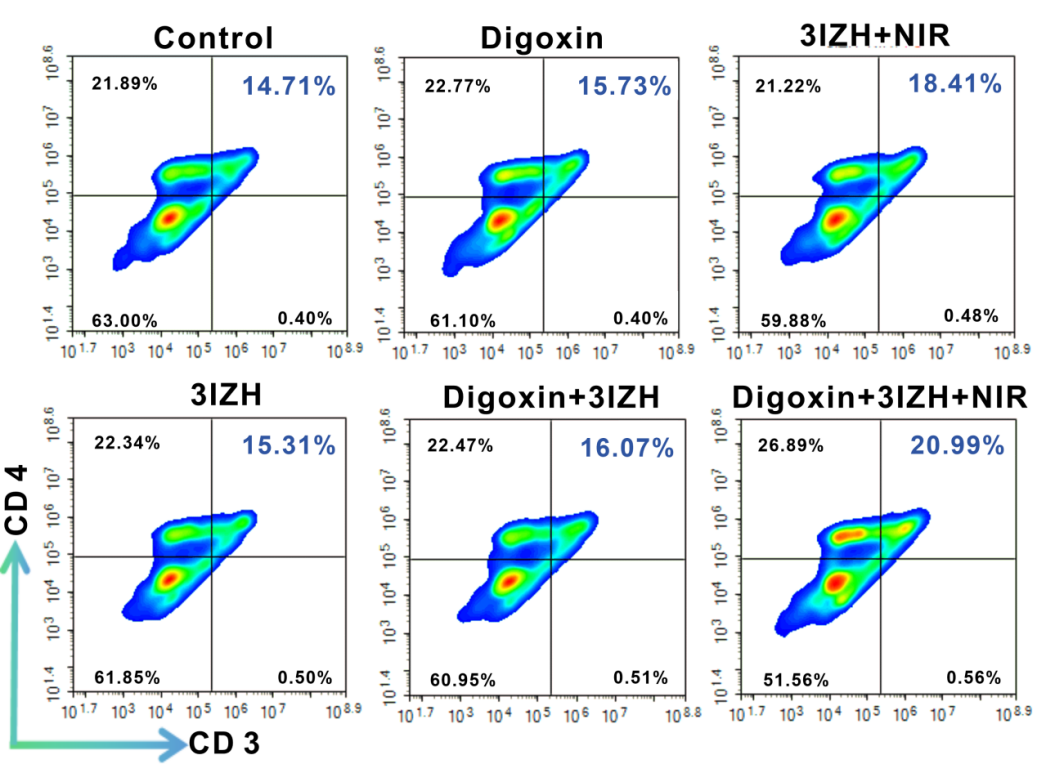


**Figure S15.** Flow cytometric detection of CD3^+^ CD4^+^ on the surface of mouse tumors (distant tumors). (mean ± SD, n = 3).


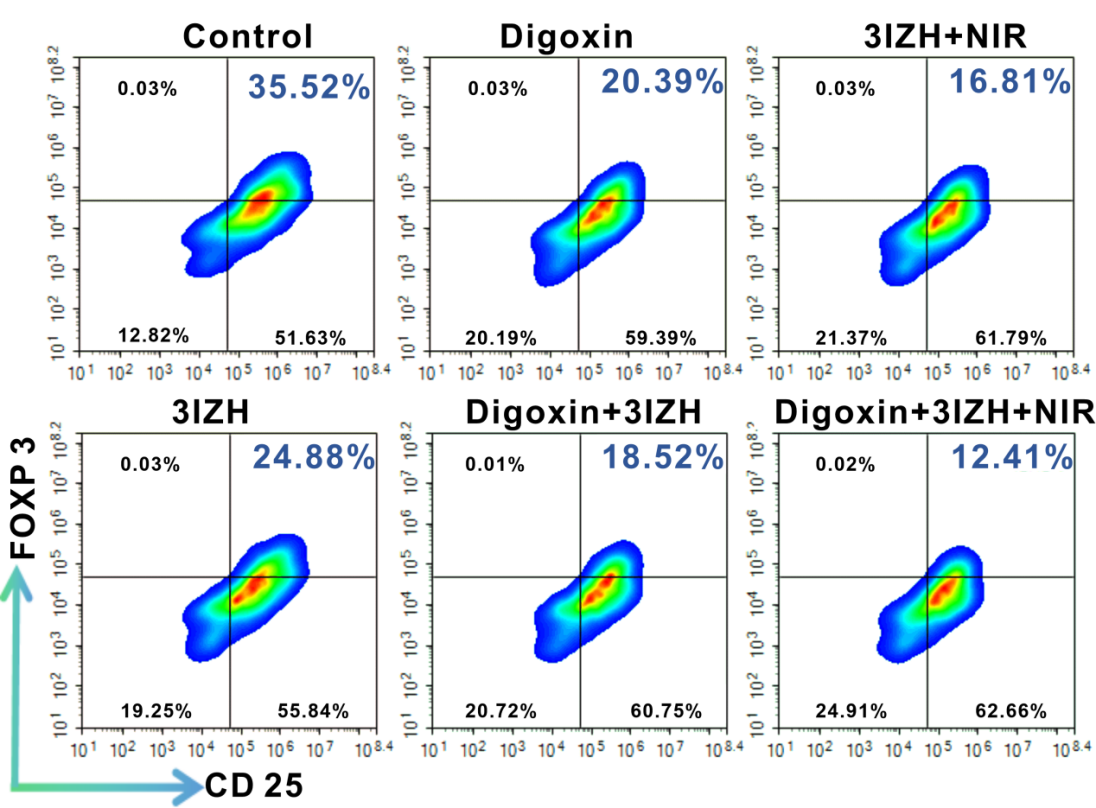


**Figure S16.** Flow cytometric detection of CD25^+^ FOXP3^+^ on the surface of mouse tumors (distant tumors). (mean ± SD, n = 3).


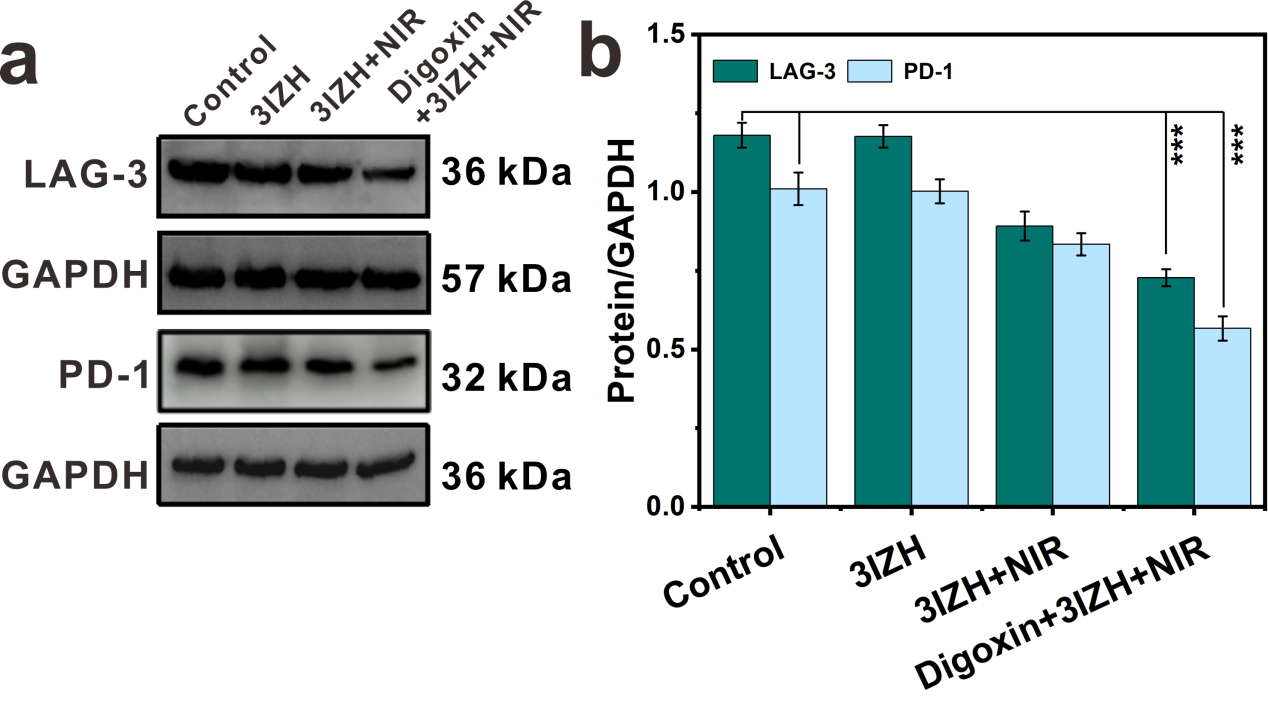


**Figure S17.** (a) The representative immunoblotting depicts the protein expression levels of the groups following different treatments. (b) Quantitative analysis of the target protein. (mean ± SD, n = 3, and *p < 0.05, **p < 0.01 and ***p < 0.001). One-way ANOVA analysis of variance was used for analysis of statistical significance.


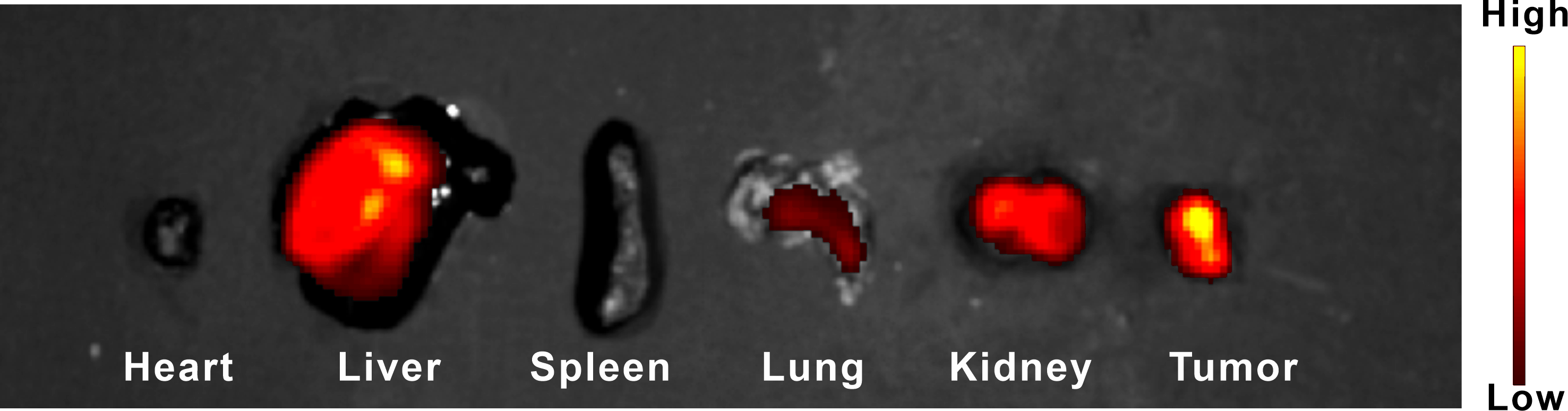


**Figure S18.** Fluorescence imaging of isolated organs in 3IZH groups at 24 h post-injection.


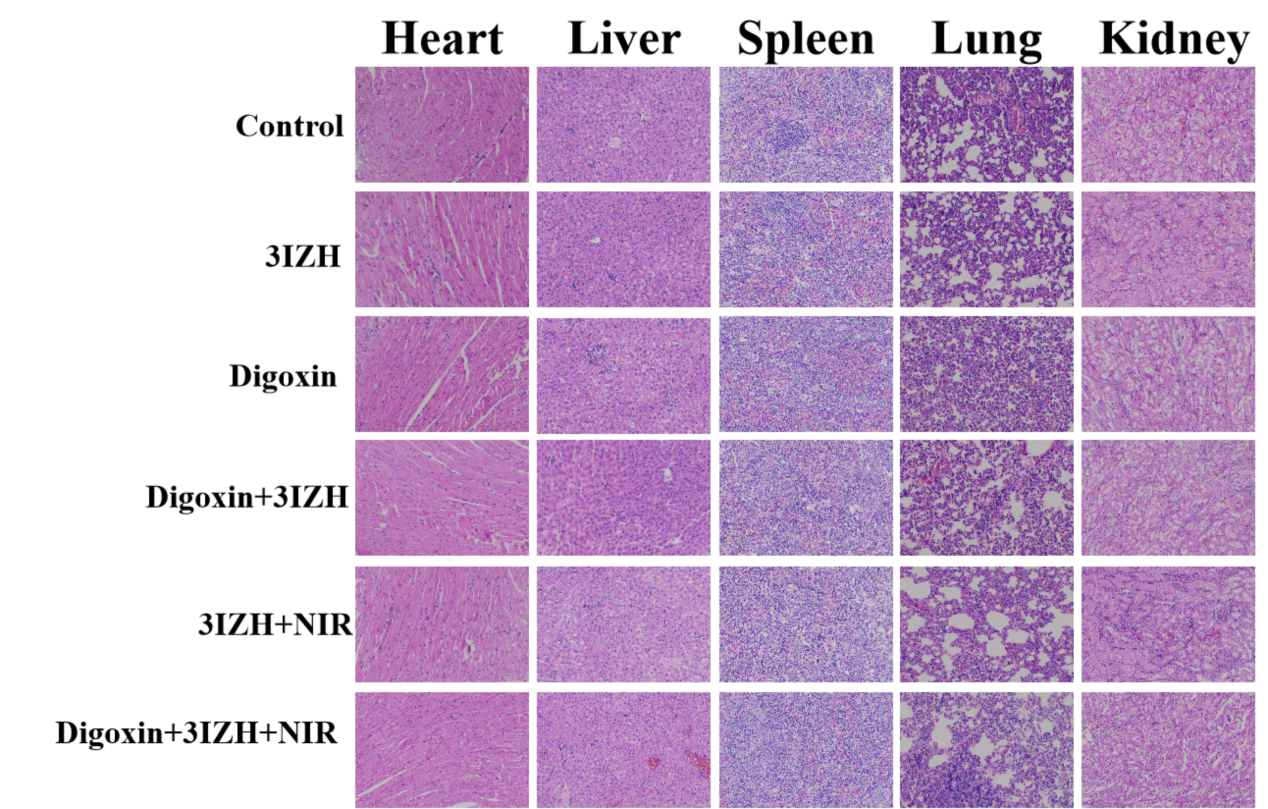


**Figure S19.** Histological staining experiments on mouse Heart, Liver, Spleen, Lung and Kidney tissues. Scale bar = 50 μm.
